# Postischemic inactivation of HIF prolyl hydroxylases in endothelium promotes maladaptive kidney repair by inducing glycolysis

**DOI:** 10.1172/JCI176207

**Published:** 2024-12-02

**Authors:** Ratnakar Tiwari, Rajni Sharma, Ganeshkumar Rajendran, Gabriella S. Borkowski, Si Young An, Michael Schonfeld, James O’Sullivan, Matthew J. Schipma, Yalu Zhou, Guillaume Courbon, Benjamin R. Thomson, Valentin David, Susan E. Quaggin, Edward B. Thorp, Navdeep S. Chandel, Pinelopi P. Kapitsinou

**Affiliations:** 1 Feinberg Cardiovascular & Renal Research Institute, and; 2 Division of Nephrology and Hypertension, Northwestern University Feinberg School of Medicine, Chicago, Illinois, USA.; 3 The Jared Grantham Kidney Institute, University of Kansas Medical Center, Kansas City, Kansas, USA.; 4 Department of Biochemistry and Molecular Genetics,; 5 Department of Pathology, and; 6 Robert H. Lurie Cancer Center, Northwestern University Feinberg School of Medicine, Chicago, Illinois, USA.

**Keywords:** Metabolism, Nephrology, Chronic kidney disease, Endothelial cells, Hypoxia

## Abstract

Ischemic acute kidney injury (AKI) is common in hospitalized patients and increases the risk for chronic kidney disease (CKD). Impaired endothelial cell (EC) functions are thought to contribute in AKI to CKD transition, but the underlying mechanisms remain unclear. Here, we identify a critical role for endothelial oxygen sensing prolyl hydroxylase domain (PHD) enzymes 1–3 in regulating postischemic kidney repair. In renal endothelium, we observed compartment-specific differences in the expression of the 3 PHD isoforms in both mice and humans. Postischemic concurrent inactivation of endothelial PHD1, PHD2, and PHD3 but not PHD2 alone promoted maladaptive kidney repair characterized by exacerbated tissue injury, fibrosis, and inflammation. scRNA-Seq analysis of the postischemic endothelial PHD1, PHD2, and PHD3-deficient (PHD^TiEC^) kidney revealed an endothelial hypoxia and glycolysis-related gene signature, also observed in human kidneys with severe AKI. This metabolic program was coupled to upregulation of the SLC16A3 gene encoding the lactate exporter monocarboxylate transporter 4 (MCT4). Strikingly, treatment with the MCT4 inhibitor syrosingopine restored adaptive kidney repair in PHDTiEC mice. Mechanistically, MCT4 inhibition suppressed proinflammatory EC activation, reducing monocyte-EC interaction. Our findings suggest avenues for halting AKI to CKD transition based on selectively targeting the endothelial hypoxia-driven glycolysis/MCT4 axis.

## Abstract 2

We demonstrate that inactivation of the endothelial oxygen sensors PHD1-3 promotes maladaptive kidney repair and identify the endothelial hypoxia-driven MCT4 as a potential therapeutic target.

## Graphical abstract

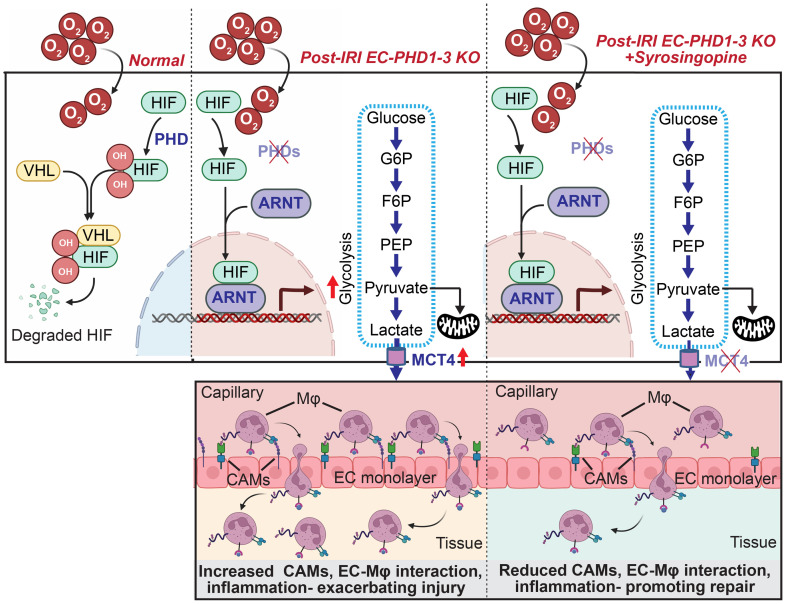

## Introduction

Ischemia is the most common cause of acute kidney injury (AKI), a condition that affects approximately 10%–15% of all hospitalized patients and more than 50% of critically ill patients (1–3). Failure to fully recover from AKI may lead to persistent functional impairment and development of chronic and progressive kidney disease, leading to end-stage renal disease (ESRD) (4). Given the lack of treatments that successfully promote kidney repair, it is crucial to better understand the mechanisms that regulate AKI to chronic kidney disease (CKD) transition and identify novel therapeutic targets.

Severe or prolonged ischemic injury can overwhelm the kidney’s reparative ability and trigger a cascade of detrimental responses within the renal microenvironment. Tubular epithelial cells undergo dedifferentiation, apoptosis, and impaired regeneration, leading to the disruption of the nephron structure (5). Immune cells, such as neutrophils and macrophages, infiltrate the injured tissue and perpetuate parenchymal cell injury and fibrosis (6). Fibroblasts become hyperactive, excessively depositing collagen and promoting tissue scarring (7). In this milieu, endothelial cells (ECs) form a metabolically dynamic barrier that, when impaired, promotes immune cell migration, dysregulated vascular tone, and permeability (8). As a result, the reduced blood flow limits oxygen delivery to affected areas leading to hypoxia. Besides the microvasculature dysfunction, the induction of hypoxia in the postischemic kidney involves multiple factors, such as increased oxygen consumption, mitochondrial dysfunction, reduced oxygen carrying capacity due to anemia, and hindered oxygen diffusion by extracellular matrix (ECM) buildup (9). While hypoxia has been linked to CKD progression, the underlying cellular mechanisms remain poorly understood.

Being in direct contact with the blood, ECs are well equipped to detect changes in oxygen levels through prolyl hydroxylase domain proteins (PHDs, also known as EGLNs), which control the stability of hypoxia-inducible factor α (HIF-α). In the presence of oxygen, PHDs hydroxylate HIF-α subunits at highly conserved proline residues promoting their ubiquitination by the von Hippel–Lindau (VHL) enzyme and ultimate proteasomal degradation (10). Under hypoxia, the reduced catalytic activity of PHDs results in HIF-α stabilization. HIF-α then translocates to the nucleus, where it dimerizes with the constitutively expressed HIF-β subunit (also known as ARNT), regulating the transcription of a broad array of genes. Genes involved in survival, metabolism, and angiogenic activity of vascular ECs are regulated by HIF with major implications in controlling vascular development and disease settings, such as cancer and inflammatory processes (11, 12). While PHD2 is considered the main oxygen sensor regulating HIF-α protein levels in normoxia, other PHD isoforms contribute to HIF regulation in particular cell types or conditions, depending on their abundance (13, 14). In vascular endothelium, remarkable heterogeneity across organs introduces an additional layer of complexity. For instance, we and others have shown a crucial role for PHD2 in regulating endothelial HIF-α in the lung, and endothelial PHD2 inactivation led to pulmonary hypertension (15, 16). On the other hand, our recent studies showed decreased responsiveness of the kidney endothelial HIF-α to PHD2 loss alone (17). Indeed, the expression of other PHD isoforms in the renal vascular bed as demonstrated by single-cell RNA sequencing (scRNA-Seq) data implied their potential contribution in regulating endothelial HIF activation in the kidney (17). Due to this complexity of the PHD/HIF system in vascular endothelium, their relevance in kidney repair after ischemic injury remain elusive.

To delineate the role of endothelial oxygen sensing in postischemic kidney repair, we have generated a set of conditional mouse strains in which we induced endothelial inactivation of PHD2 alone, or in combination with PHD1 and PHD3. While PHD2 inactivation alone did not alter kidney repair, simultaneous postischemic inactivation of endothelial PHD1, PHD2, and PHD3 induced robust HIF activation and exacerbated fibrosis, inflammation, and capillary dropout. scRNA-Seq analysis revealed a signature of endothelial hypoxia and glycolysis in association with proinflammatory responses. Loss of function of endothelial ARNT directly revealed a critical role for endothelial-derived HIF in postischemic kidney repair. Finally, using a pharmacologic approach, we identified the hypoxia-regulated lactate exporter monocarboxylate transporter 4 (MCT4) as a potential target to suppress maladaptive proinflammatory responses and halt AKI to CKD transition.

## Results

### Postischemic inactivation of endothelial PHD2 does not affect postischemic kidney injury.

Our prior studies have shown that constitutive or acute inactivation of PHD2 in ECs prior to kidney ischemia-reperfusion injury (IRI) prevents postischemic injury and inflammation (17, 18). While these findings show the beneficial effects of endothelial PHD/HIF signaling to promote kidney resilience against ischemic insult, they cannot be extrapolated to kidney repair following established injury, in which distinct responses control regeneration of the damaged tissue (19). To ask whether inhibition of endothelial PHD2 regulates kidney repair, we used *Cdh5-CreER^T2^;Phd2^fl/fl^* mice (*PHD2^iEC^*) (17, 20), which allow induction of recombination following established kidney injury. We first assessed the endothelial recombination efficiency achieved when tamoxifen treatment started on day 1 after renal unilateral IRI (uIRI) and included 4 i.p. doses given every other day, as indicated in Supplemental Figure 1A (supplemental material available online with this article; https://doi.org/10.1172/JCI176207DS1). Using *Cdh5(PAC)CreER^T2^-Rosa26-mTmG* mice (17), we performed flow cytometry (Supplemental Figure 1B) and found that approximately 100% of GFP^+ve^ cells in day 14 postischemic kidneys were also CD31^+ve^. This recombination efficiency was comparable to the contralateral kidneys. Otherwise, at day 14 after IRI, there was an approximate 50% reduction in kidney ECs, which is expected in the setting of IRI-induced capillary rarefaction (Supplemental Figure 1C).

**Figure 1:**
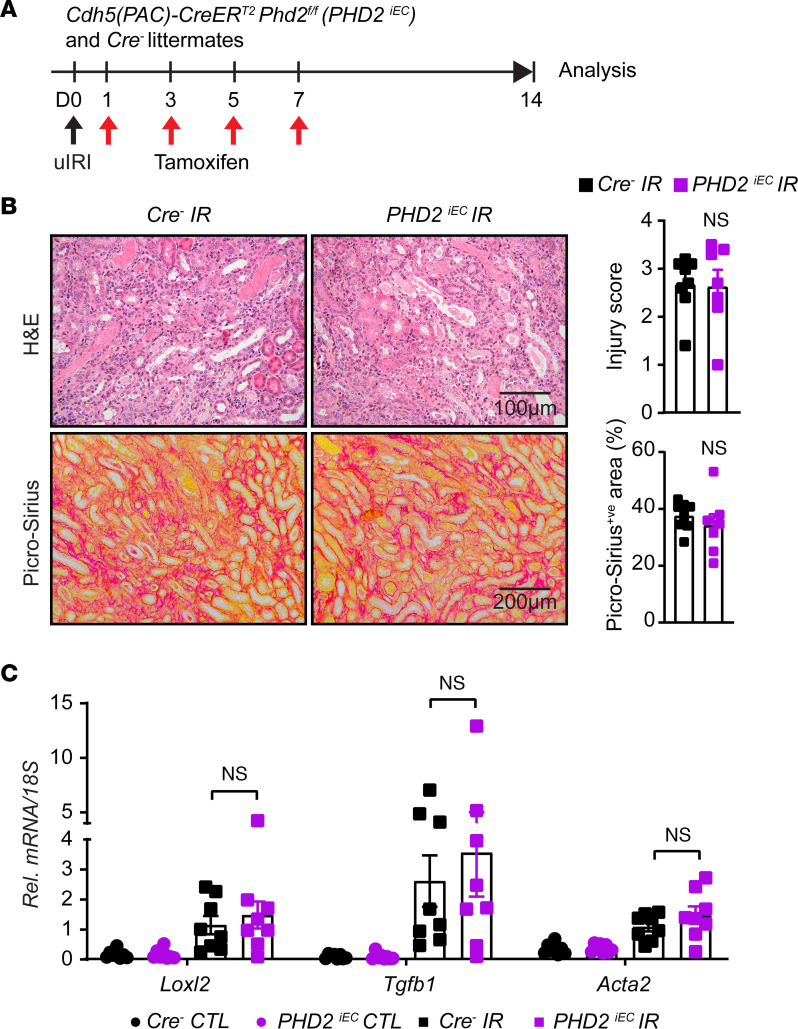
Postischemic inactivation of endothelial PHD2 does not alter postischemic kidney injury. (**A**) Experimental schematic illustrates the timing of unilateral renal artery clamping, tamoxifen administration, and analysis. (**B**) Representative images of H&E- and Picrosirius red– stained sections from day 14 postischemic kidneys of *PHD2^iEC^* mutants and their *Cre^–^* littermates. Right panels show tubular injury score (top) and semiquantitative analysis of Picrosirius red^+ve^ area in the indicated genotypes. Scale bars: 100 μm (H&E); 200 μm (Picrosirius red). (**C**) mRNA levels of *Loxl2*, *Tgfb1*, and *Acta2* in IR and CTL kidneys from *PHD2^iEC^* mice and their *Cre^–^* controls at day 14 after uIRI. All bars show mean ± SEM. For **B**, unpaired *t* test with Welch’s correction was used. For **C**, statistics were determined using 1-way ANOVA with Šidák’s correction for multiple comparisons. *n* = 7–8. CTL, contralateral; IR, kidney subjected to ulRI; Rel., relative.

After validating the recombination efficiency of the *Cdh5(PAC)CreER^T2^* system in the postischemic kidney, adult male *PHD2^iEC^* mice and their *Cre^–^* littermates were subjected to uIRI, followed by tamoxifen treatment and analysis on day 14, as indicated in Figure 1A. Assessment of tubular injury on H&E-stained sections of day 14 postischemic kidneys revealed that *PHD2^iEC^* showed comparable tubular damage to *Cre^–^* littermate controls. Furthermore, quantitative analysis of Picrosirius red staining showed no significant difference in collagen accumulation between mutants and controls (Figure 1B). Finally, day 14 postischemic kidneys showed significantly increased transcripts of profibrotic genes lysyl oxidase-like 2 (*Loxl2*), transforming growth factor β 1 (*Tgfb1*), and smooth muscle cell a-actin (*Acta2*) compared with their corresponding contralaterals, but there was no significant change between the 2 genotypes (Figure 1C). Therefore, acute postischemic endothelial inactivation of PHD2 does not affect kidney repair and transition to CKD following IRI.

### Kidney endothelium shows compartment-specific differential expression of PHD1, -2, and -3 in mice and humans.

**Figure 2:**
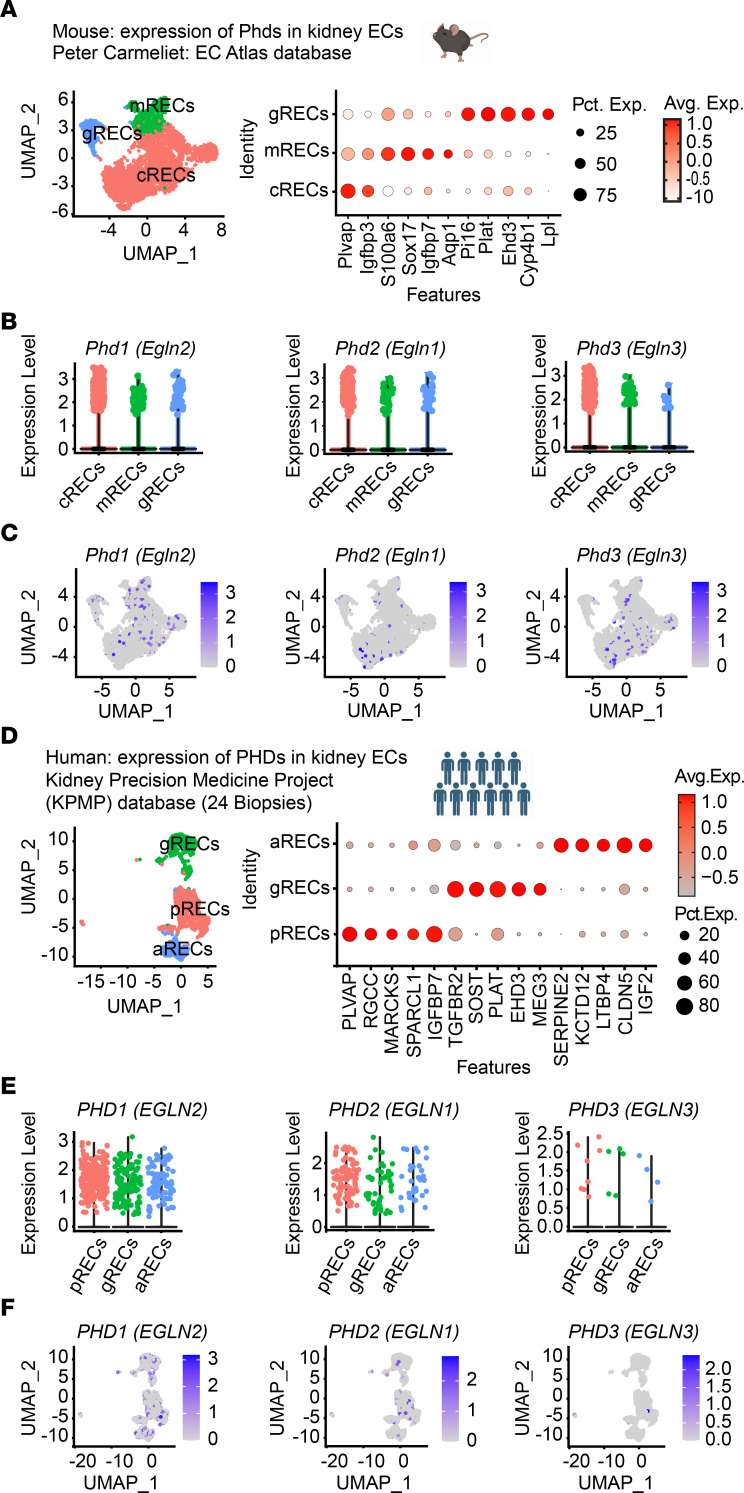
scRNA-Seq analysis shows differential expression of *PHD1, PHD2,* and *PHD3* in kidney ECs in mice and humans. (**A**–**C**) scRNA-Seq analysis of RECs extracted from mouse EC Atlas database (https://endotheliomics.shinyapps.io/ec_atlas/). (**A**) UMAP plot shows 3 EC clusters: cRECs, mRECs, and glomerular RECs (gRECs). Dot plot displays gene expression patterns of cluster-enriched markers. Violin plots (**B**) and feature plots (**C**) show the expression of *Phd1* (*Egln2*), *Phd2*, (*Egln1*) and *Phd3* (*Egln3*) in murine RECs. (**D**–**F**) scRNA-Seq analysis of RECs extracted from normal human kidney biopsies (*n* = 24). (**D**) UMAP plot shows arteriolar RECs (aRECs), glomerular RECs, and peritubular RECs (pRECs). Dot plot illustrates gene expression patterns of cluster-enriched markers. Violin plots (**E**) and feature plots (**F**) show the expression of *PHDs* in different REC clusters.

We recently reported that inactivation of endothelial PHD2 alone was not sufficient to stabilize HIF in the renal endothelium (17), raising the possibility for important contributions of PHD1 and/or PHD3 in regulating renal endothelial HIF protein levels. To better understand the role of oxygen sensors in renal vasculature, we took advantage of publicly available scRNA-Seq data and characterized the expression of different *Phd* isoforms in kidney EC. We first analyzed the expression of *Phd1* (*Egln2)*, *Phd2* *(Egln1)*, and *Phd3* *(Egln3)* in scRNA-Seq data of renal ECs (RECs) extracted from the murine EC Atlas database (https://endotheliomics.shinyapps.io/ec_atlas/) (21) (Figure 2, A–C). We found that *Phd1* was ubiquitously expressed, whereas *Phd2* and *Phd3* were mainly expressed in cortical RECs (cRECs) and medullary RECs (mRECs). Glomerular RECs showed particularly low expression for *Phd3*. To further validate the differential expression of PHDs in murine kidney endothelium, we immunostained kidney sections from tamoxifen-treated *Cdh5(PAC)CreER^T2^-Rosa26-mTmG* reporter mice with antibodies against PHD1, PHD2, and PHD3. Immunolabeling confirmed ubiquitous expression of PHD1, expression of PHD2 in cRECs and mRECs, and low expression of PHD3 in glomerular RECs (Supplemental Figure 2).

Next, to characterize the expression of PHD isoforms within human kidney endothelium, we analyzed scRNA-Seq data of human kidneys available through the Kidney Precision Medicine Project (KPMP) (Figure 2, D–F, and Supplemental Table 1) (22). ECs were identified by the expression of *CD34* and endomucin (*EMCN*) and were clustered to 3 subclusters based on the expression of previously validated specific markers: *PLVAP* in peritubular ECs, *EHD3* in glomerular, and *SERPINE2* in arteriolar ECs (Figure 2D) (22). This analysis demonstrated that *PHD1* was the most highly expressed isoform whereas *PHD3* showed the lowest expression. Among the different compartments, *PHD2* was predominantly expressed in peritubular capillary ECs (Figure 2, E and F). Together, these studies suggest endothelial compartment–specific differences in the expression of the 3 PHD isoforms in mice and humans.

### Postischemic concurrent inactivation of endothelial PHD1, -2, and -3 promotes HIF activation and maladaptive kidney repair.

Because we found that all PHD isoforms are expressed in kidney endothelium with variable abundance within different compartments, we next generated mice that allow acute endothelial specific inactivation of PHD1, PHD2, and PHD3 (*Cdh5(PAC)CreER^T2^;* *Phd1^fl/fl^* *Phd2^fl/fl^* *Phd3^fl/fl^* *mice*; *PHD^TiEC^*) and predicted that the resulting mutants would show HIF stabilization in endothelium. One week after the completion of tamoxifen treatment (Supplemental Figure 3A), coimmunostaining with antibodies against PHD1, -2, and -3 and EMCN demonstrated successful ablation of PHD1, -2, and -3 in kidney ECs of *PHD^TiEC^* compared with *Cre^–^* littermates (Supplemental Figure 3B). Further, immunoblot assessment of kidney nuclear extracts revealed significant stabilization of HIF-1α and HIF-2α in the kidneys of *PHD^TiEC^* mice (Supplemental Figure 3C). Baseline histopathological analysis showed normal kidney morphology without fibrosis in *PHD^TiEC^* mice as indicated by H&E and Picrosirius red staining (Supplemental Figure 3D).

**Figure 3:**
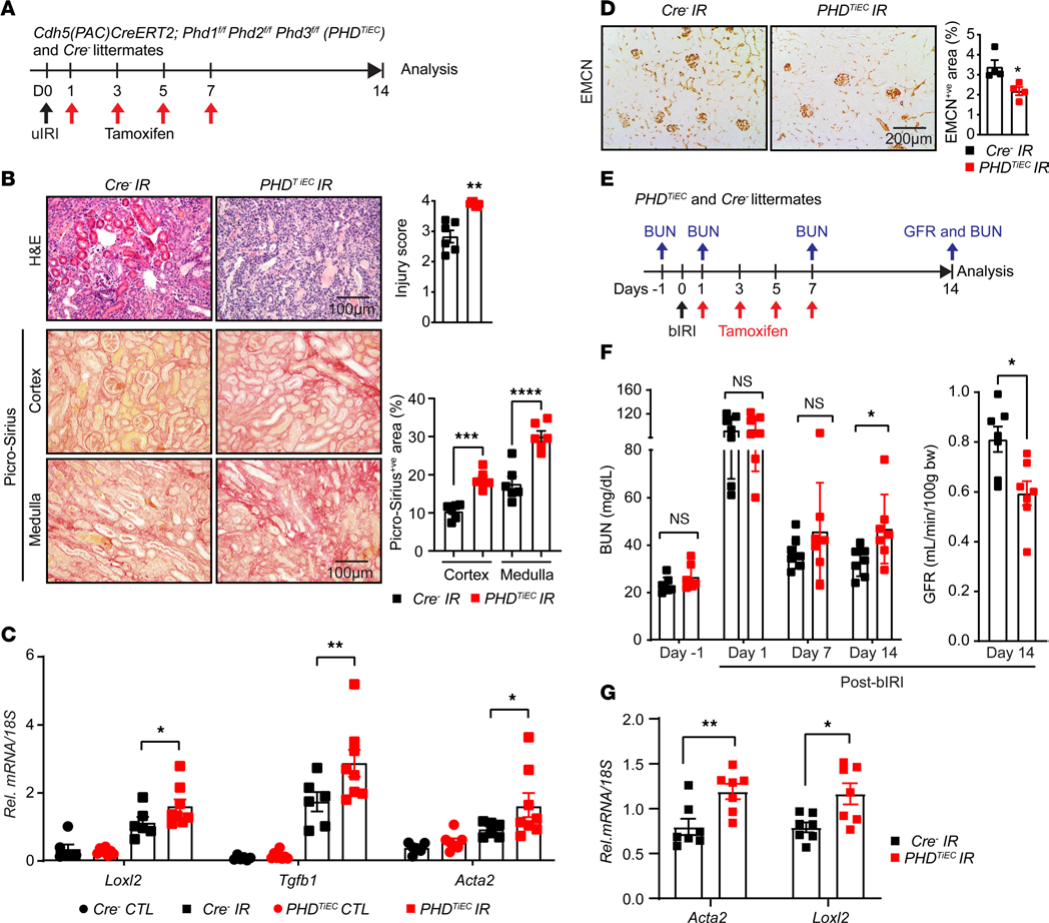
Postischemic simultaneous inactivation of endothelial PHD1, -2, and -3 promotes maladaptive kidney repair. (**A**) Schematic illustrating the experimental strategy applied for uIRI studies. (**B**) Representative images of H&E- and Picrosirius red–stained sections as well as tubular injury score and semiquantitative analysis of Picrosirius red^+ve^ area on day 14 postischemic kidneys from *PHD^TiEC^* mice and *Cre^–^* littermates. Scale bars: 100 μm (H&E); 200 μm (Picrosirius red). (**C**) mRNA levels of *Loxl2*, *Tgfb1*, and *Acta2* in IR and CTL kidneys from *PHD^TiEC^* mice and their *Cre^–^* controls at day 14 after uIRI. (**D**) Representative images of EMCN immunostaining and semiquantitative analysis of EMCN^+ve^ peritubular capillary area on day 14 postischemic kidneys from *PHD^TiEC^* mice and *Cre^–^* littermates. (**E**) Schematic depicting the experimental workflow for bIRI studies. (**F**) Serum BUN levels at different time points and GFR measurements on day 14 after bIRI. All bars show mean ± SEM of each group. (**G**) mRNA levels of *Acta2* and *Loxl2* in IR kidneys from PHD*^TiEC^* mice and their *Cre^–^* controls at day 14 after bIRI. For **B**, **D**, **F**, and **G**, statistics were determined by unpaired *t* test with Welch’s correction. For **C**, 1-way ANOVA with Šidák’s correction for multiple comparisons was used. *P < 0.05; **P < 0.01; ***P < 0.001; ****P < 0.0001. *n* = 4–8.

To determine the impact of postischemic inactivation of endothelial PHD1, PHD2, and PHD3 on kidney repair following IRI, *Cre^–^* and *PHD^TiEC^* adult male mice were subjected to unilateral renal artery clamping for 25 minutes followed by tamoxifen treatment and analysis on day 14 (Figure 3A). Notably, histological analysis of day 14 postischemic kidneys revealed higher tubular injury scores in *PHD^TiEC^* mice than in *Cre^–^* controls (Figure 3B). Furthermore, day 14 postischemic kidneys from *PHD^TiEC^* mutants showed approximately 79% and 70% increase in collagen deposition in cortex and medulla, respectively, compared with *Cre^–^* littermates as indicated by Picrosirius red staining (Figure 3B). Increased fibrosis in the postischemic *PHD^TiEC^* mutant kidneys was also confirmed by Masson’s trichrome staining (Supplemental Figure 4A) and immunofluorescence staining for α-smooth muscle actin (aSMA) (Supplemental Figure 4B). Accordingly, quantitative PCR (qPCR) analysis showed significant transcriptional induction of profibrotic genes *Loxl2*, *Tgfb1*, and *Acta2*, indicating increased postischemic kidney fibrosis in *PHD^TiEC^* mutants compared with *Cre^–^* controls (Figure 3C). Because peritubular capillary rarefaction is a hallmark of fibrotic kidneys, we also assessed capillary density by EMCN staining and found that the EMCN^+ve^ area was reduced by 35% in day 14 postischemic kidneys from *PHD^TiEC^* mice (Figure 3D) (23–26). On the other hand, female *PHD^TiEC^* mice subjected to uIRI demonstrated no significant increase in tubular injury and fibrosis compared with their female *Cre^–^* littermates (Supplemental Figure 5), despite increasing clamping time to 30 minutes to achieve consistent injury. Sex differences in hypoxia responses (27, 28) or the effects of tamoxifen (29) may play a role in this variation in females versus males. Hereafter, experiments were conducted exclusively on male mice.

To assess the impact of postischemic endothelial inactivation of PHD1, PHD2, and PHD3 on recovery of kidney function after IRI, *PHD^TiEC^* and *Cre^–^* littermate mice were subjected to bilateral renal IRI (bIRI). In this model, both renal arteries were clamped, but clamping time was reduced to 23 minutes to allow survival of mice. As shown in Figure 3E, tamoxifen was started on day 1 after bIRI while blood urea nitrogen (BUN) levels were measured 1 day prior to surgery (baseline) and on days 1, 7, and 14 after bIRI. As expected, on day 1 after bIRI, BUN levels increased to a similar degree for both genotypes without a significant difference on day 7. However, on day 14 after bIRI, *PHD^TiEC^* had significantly increased BUN levels by 43% compared with *Cre^–^* mice (*P*<0.05). In accordance, glomerular filtration rate (GFR) measurements on day 14 after bIRI showed a 26% reduction in GFR of *PHD^TiEC^* mice compared with their *Cre^–^* littermates (Figure 3F). The impaired recovery of renal function in *PHD^TiEC^* mice was associated with increased expression of *Acta2* and *Loxl2* mRNAs in day 14 post-bIRI kidneys compared with their controls (Figure 3G). In conclusion, our findings from 2 models of renal IRI demonstrate that EC-specific inactivation of PHD1, PHD2, and PHD3 after ischemic injury promotes tubular injury, interstitial fibrosis, and capillary drop-out, leading to impaired recovery of kidney function.

### scRNA-Seq reveals distinct cell-specific responses in postischemic PHD^TiEC^ mutant kidney.

**Figure 4:**
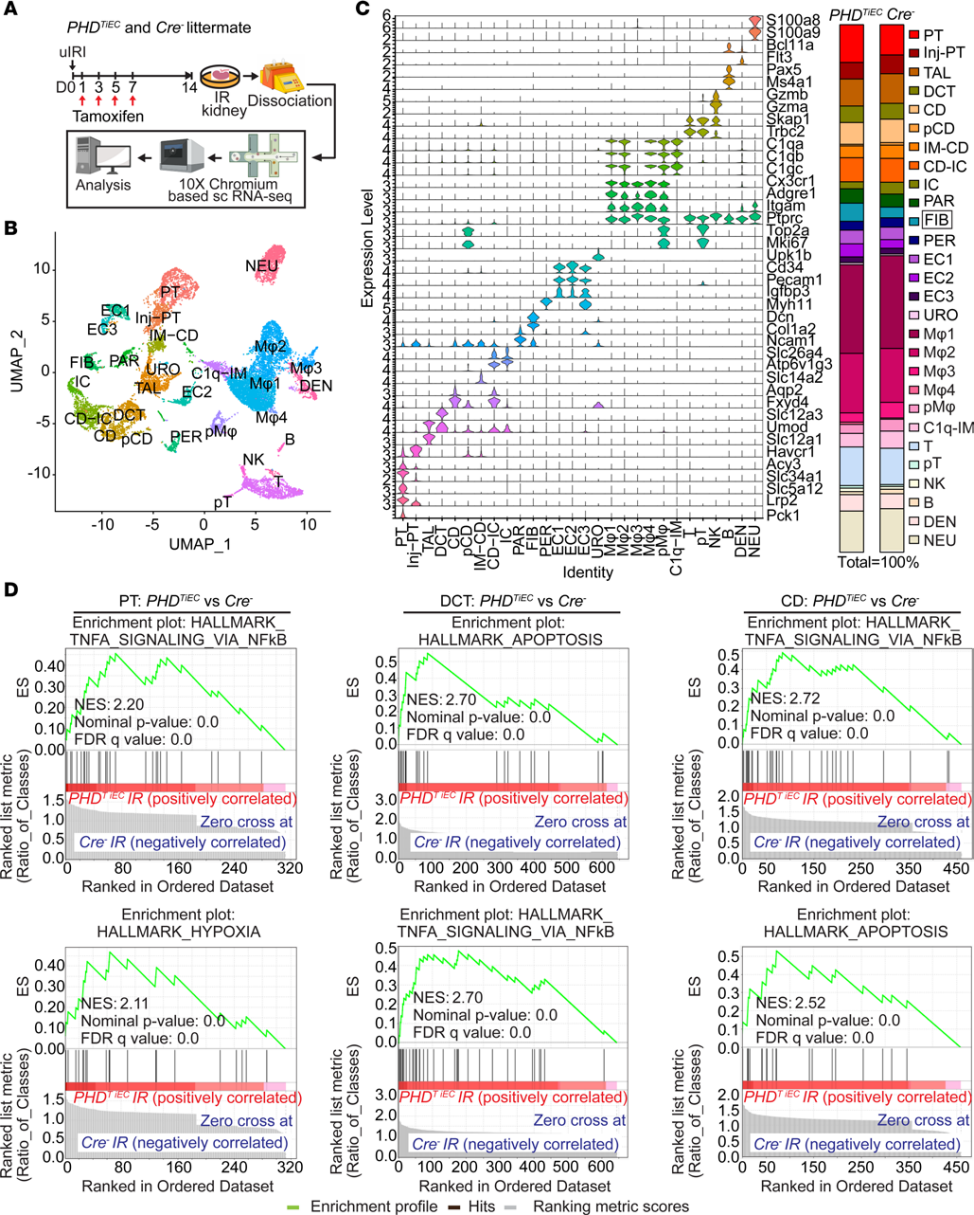
scRNA-Seq analysis reveals the cellular landscape of day 14 postischemic kidneys of *PHD^TiEC^* and control mice. (**A**) Schematic illustrating the experimental strategy applied for scRNA-Seq analysis. A *PHD^TiEC^* mouse and a *Cre^–^* littermate were subjected to 25 minutes of unilateral renal artery clamping. Treatment with tamoxifen was started on day 1 post uIRI and involved 4 i.p. doses given every other day. Mice were sacrificed for scRNA-Seq analysis on day 14 after uIRI. IR kidneys were isolated and single-cell suspension was prepared and used for scRNA-Seq analysis (*n* = 1 per genotype). (**B**) UMAP plot representation of the cell classification in day 14 postischemic kidneys from *PHD^TiEC^* and *Cre^–^* mice. (**C**) Violin plots display characteristic marker genes for each identified cell population. Right side bar plot shows cell proportions in day 14 postischemic kidneys from *Cre^–^* and *PHD^TiEC^* mice. Highlighted is the increased proportion of FIBs in *PHD^TiEC^* postischemic kidney compared with *Cre^–^* control. PT, Inj-PT, TAL, DCT, CD, pCD, IM-CD, CD-IC, IC, PAR, FIB, PER, EC1-3, URO, Mφ1-4, pMφ, C1q-IM, T, pT, NK, B, DEN, and NEU. (**D**) Top 2 enriched Hallmark pathways emerged in GSEA hallmark analysis of DEGs for PT, DCT, and CD clusters of *PHD^TiEC^* postischemic kidney as compared with *Cre^–^* control.

To investigate the impact of postischemic endothelial PHD inactivation on cell-type–specific alterations in the context of kidney repair following IRI, we carried out scRNA-Seq on day 14 postischemic kidneys from *PHD^TiEC^* and *Cre^–^* control mice. Using the 10X Genomics Chromium platform, we aimed for 10,000 cells per group (Figure 4A). After quality filtering, a total of 16,698 (7,955 from *Cre^–^* and 8,743 from *PHD^TiEC^*) cells remained for subsequent analysis. We identified 28 different cell clusters using unsupervised graph-based clustering followed by principal component analysis (PCA) and uniform manifold approximation and projection (UMAP) (Figure 4B). When we overlayed *Cre^–^* ischemia-reperfusion (IR) and *PHD^TiEC^* IR samples, we observed similar cell clusters in each genotype (Supplemental Figure 6). Based on expression of reported canonical marker genes (Supplemental Table 2), we identified all expected kidney cell types: proximal tubule (PT), injured PT (Inj-PT), thick ascending limb (TAL), distal convoluted tubule (DCT), collecting duct (CD), proliferating CD (pCD), inner medullary collecting duct (IM-CD), collecting duct-intercalated cells (CD-IC), intercalated cells (IC), parietal cells (PAR), fibroblasts (FIB), pericytes (PER), ECs 1-3 (EC1-3), urothelial cells (URO), macrophages 1-4 (Mφ1-4), proliferating macrophages (pMφ), C1q (C1qa, C1qb and C1qc) expressing immune cells (C1q-IM), T cells (T), proliferating T cells (pT), NK cells, B cells (B), dendritic cells (DEN), and neutrophils (NEU) (Figure 4C). Consistent with the aforementioned increased fibrosis, analysis of the relative cell proportions for each genotype showed increased FIBs by approximately 1.9-fold in the postischemic *PHD^TiEC^* kidney compared with control. Next, we performed differential gene expression analysis for tubular clusters between the 2 genotypes. After applying cutoff criteria of a log_2_ fold change >0.2 and adjusted *P* < 0.05, PT, Inj-PT, TAL, DCT, CD, and CD-IC clusters of *PHD^TiEC^* IR kidney showed 163, 90, 209, 269, 211, and 250 differentially expressed genes (DEGs), respectively (Supplemental Data File 1). Gene set enrichment analysis (GSEA) demonstrated substantial differences in tubular cells between *PHD^TiEC^* and *Cre^–^*. Within the tubular clusters, the top Hallmark gene signatures were tumor necrosis factor (TNFA) signaling via NF-κB and hypoxia pathways for PT; apoptosis, TNFA signaling via NF-κB, and hypoxia pathway for DCT; and TNFA signaling via NF-κB, apoptosis, and hypoxia pathway for CD (Figure 4D and Supplemental Data File 2). Overall, these results show that the exacerbated fibrotic response in postischemic kidneys of *PHD^TiEC^* mutants is associated with a proinflammatory signature in tubular cells.

### mRECs of postischemic PHD^TiEC^ mutant kidney show increased glycolysis.

**Figure 5:**
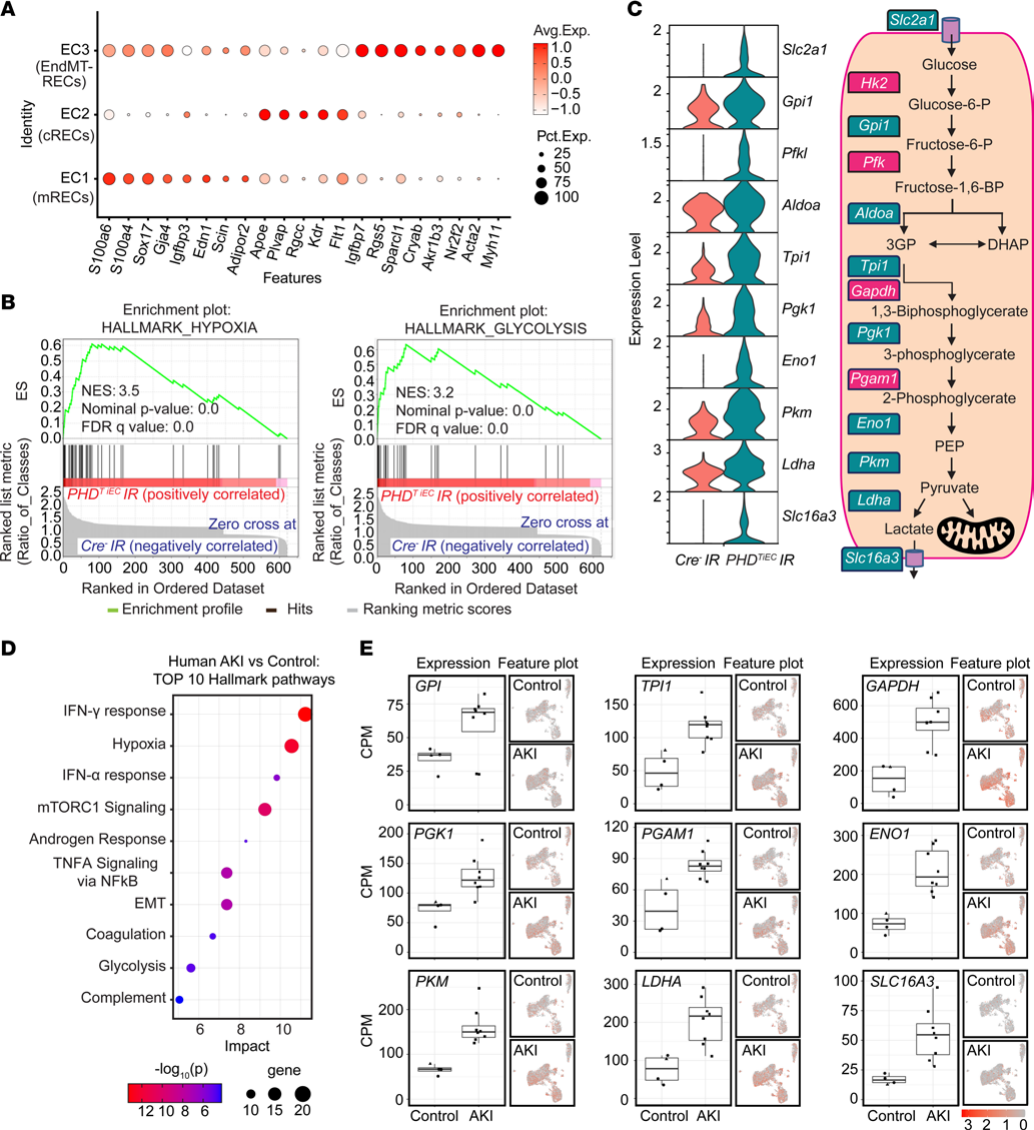
Postischemic endothelial PHD inactivation induces a hypoxia and glycolysis gene signature in mRECs. (**A**) Dot plot visualization shows the expression of marker genes used to identify cRECs, mRECs, and EndMT-RECs clusters. (**B**) GSEA in mRECs of *PHD^TiEC^* kidney compared with control. Among the most highly enriched Hallmark pathways were hypoxia and glycolysis. (**C**) Violin plots show significantly upregulated glycolytic genes in mRECs of *PHD^TiEC^* compared with control. Pathway diagram summarizes the functions of upregulated genes (marked by teal boxes) in glycolysis. (**D** and **E**) snRNA-Seq analysis of human kidney tissue from patients with severe AKI and controls (*n* = 6–8). Analysis was performed on publicly available snRNA-Seq data from Christian Hinze et al. (34). (**D**) Bubble chart for top 10 enriched Hallmark pathways of upregulated DEGs in kidney ECs from patients with severe AKI compared with controls. (**E**) Box plots show the expression of glycolytic genes in kidney ECs in controls versus AKI patients. The expression levels of glycolytic genes were extracted from the online interface provided by Christian Hinze et al. (https://shiny.mdc-berlin.de/humAKI). CPM, normalized counts per million.

Next, we focused on the 3 EC clusters annotated as mRECs, cRECs, and RECs expressing mesenchymal markers (EndMT-RECs) based on marker genes established from the Carmeliet group (30) and others (31–33) (Figure 5A and Supplemental Figures 7 and 8). Among the EC clusters, mRECs showed the majority of DEGs between the 2 genotypes (58 genes with adjusted *P* value < 0.05) (Supplemental Data File 3), while the numbers of DEGs for cRECs and EndMT-RECs were more limited (2 and 12 genes respectively). GSEA analysis of DEGs in mRECs identified hypoxia and glycolysis as the top 2 significantly enriched Hallmark pathways followed by MTORC1 signaling and epithelial mesenchymal transition pathways in *PHD^TiEC^* compared with *Cre^–^* mouse (Figure 5B and Supplemental Data File 3). Consistent with enhanced glycolytic activity, the glycolysis-related genes solute carrier family 2 (facilitated glucose transporter), member 1 (*Scl2a1*), glucose-6-phosphate isomerase 1 (*Gpi1*), phosphofructokinase, liver, B-type (*Pfkl*), aldolase A, fructose-bisphosphate (*Aldoa*), triosephosphate isomerase 1 (*Tpi1*), phosphoglycerate kinase 1 (*Pgk1*), enolase 1, α nonneuron (*Eno1*), pyruvate kinase, muscle (*Pkm*), lactate dehydrogenase A (*Ldha*), and solute carrier family 16, member 3 (*Slc16a3*) were significantly upregulated in mRECs of *PHD^TiEC^* IR kidney compared with *Cre^–^* control (Figure 5C).

To examine whether this metabolic profile in ECs was relevant to human AKI, we analyzed publicly available single nucleus RNA sequencing (snRNA-Seq) data from kidney tissues of patients with severe AKI (34). Hallmark analysis showed hypoxia and glycolysis among the top enriched pathways in ECs of human kidneys with AKI (Figure 5D and Supplemental Data File 4). Specifically, we found upregulation of glucose-6-phosphate isomerase (*GPI*), triosephosphate isomerase 1 (*TPI1*), glyceraldehyde-3-phosphate dehydrogenase (*GAPDH*), phosphoglycerate kinase 1 (*PGK1*), phosphoglycerate mutase 1 (*PGAM1*), enolase 1 (*ENO1*), pyruvate kinase M1/2 (*PKM*), lactate dehydrogenase A (*LDHA*), and solute carrier family 16 member 3 (*SLC16A3*) in ECs from kidney tissues of patients with AKI compared with the control group (Figure 5E). Overall, these results demonstrate that postischemic endothelial inactivation of PHDs activates the hypoxia response, leading to increased endothelial glycolysis, a metabolic response that is also observed in patients with severe AKI.

### Postischemic endothelial inactivation of PHD1, PHD2, and PHD3 induces EC-derived proinflammatory responses and exacerbates macrophage accumulation.

**Figure 6:**
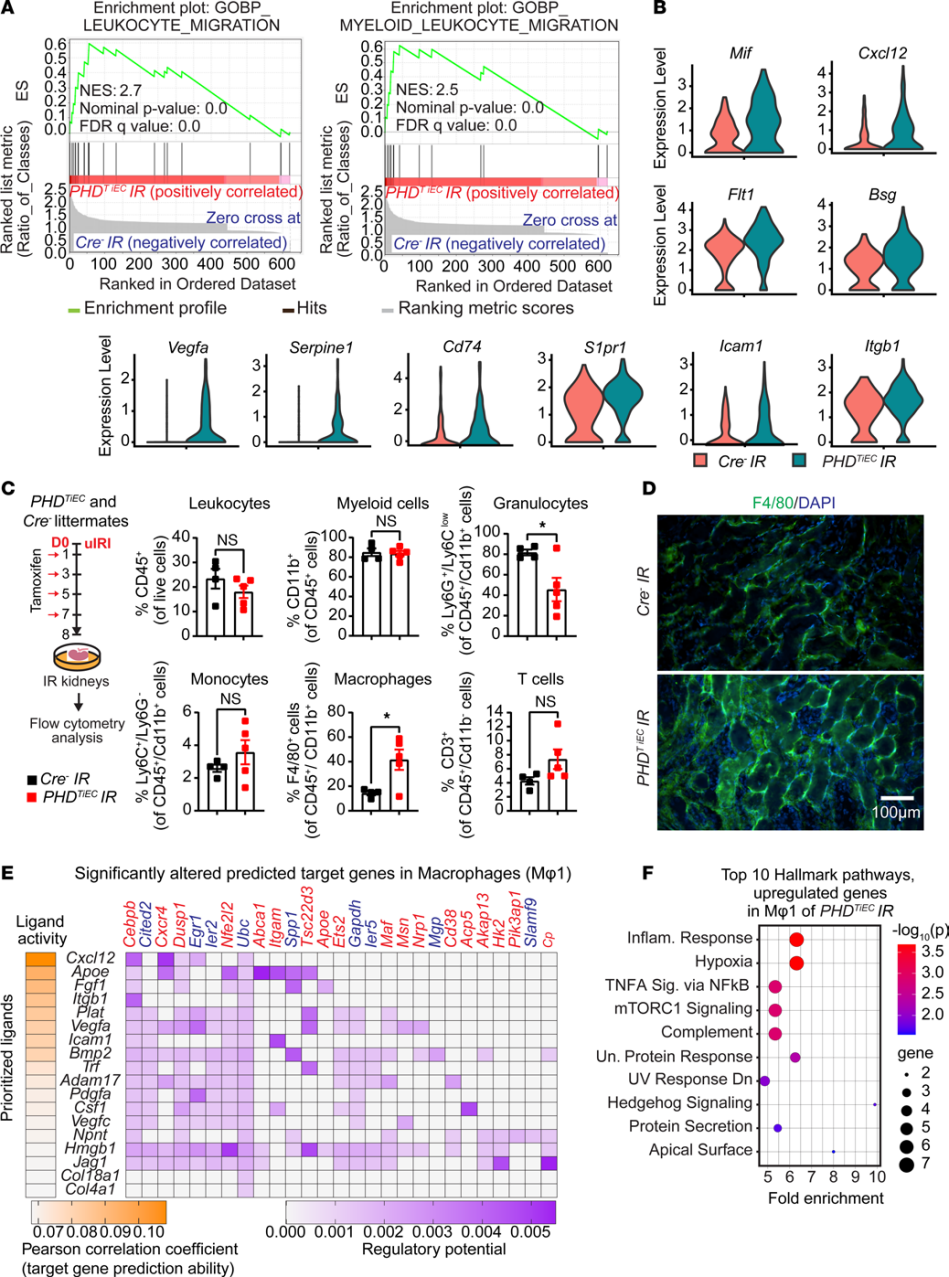
Postischemic inactivation of endothelial PHDs induces EC-derived proinflammatory responses. (**A**) GSEA plots show significant enrichment for GO-biological processes (GOBP) of leukocyte migration and myeloid leukocyte migration in mRECs of *PHD^TiEC^* postischemic kidney compared with control. (**B**) Violin plots display the expression levels of proinflammatory genes associated with core enrichment in GOBP-leukocyte migration and myeloid leukocyte migration in mRECs of *PHD^TiEC^* compared with control. (**C**) Shown is the experimental strategy for flow cytometry analysis. Eight days after uIRI, postischemic kidneys from *PHD^TiEC^* and *Cre^–^* control mice were harvested, and flow cytometry analysis of immune cells was performed (*n* = 4–5). Data are represented as mean ± SEM. Statistics were determined by unpaired *t* test with Welch’s correction. (**D**) Representative images of immunofluorescence staining for F4/80 (green) and nuclear DAPI staining (blue) of day 8 postischemic kidneys from *PHD^TiEC^* and *Cre^–^* control mice. Images were captured using a Nikon Ti2 Widefield fluorescence microscope. Scale bar: 100 μm. (**E**) Shown is NicheNet analysis of mRECs communication with macrophage Mφ1 cluster in day 14 post-IRI kidney of *PHD^TiEC^* mutant compared with control. Top prioritized ligands expressed by mRECs (senders) and target genes that are significantly altered (red, upregulated genes; blue, downregulated genes) in the macrophages Mφ1 (receivers). The interaction pairs were derived from the NicheNet data sources and analysis. (**F**) Hallmark analysis of significantly upregulated genes in Mφ1 cluster of *PHD^TiEC^* kidney compared with *Cre^–^* control. Top 10 pathways are shown. ^*^P < 0.05.

GSEA analysis of DEGs is mRECs showed high enrichment of Gene Ontology Biological Process (GOBP) gene sets of leukocyte migration and myeloid leukocyte migration. These gene signatures were driven by upregulation of macrophage migration inhibitory factor (*Mif*), C-X-C motif chemokine ligand 12 (*Cxcl12*), FMS-like tyrosine kinase 1 (*Flt1*), basigin (*Bsg*), vascular endothelial growth factor A (*Vegfa*), serine (or cysteine) peptidase inhibitor, clade E, member 1 (*Serpine1*), CD74 antigen (*Cd74*), sphingosine-1-phosphate receptor 1 (*S1pr1)*, intercellular adhesion molecule 1 (*Icam1*), and integrin β 1 (*Itgb1*) in *PHD^TiEC^* IR kidney compared with control (Figure 6, A and B). Using immunofluorescence staining, we confirmed the increased expression of CXCL12 and ICAM1 in the kidney endothelium of *PHD^TiEC^* mutants (Supplemental Figure 9). To characterize immune cell alterations in the postischemic kidney microenvironment in the context of endothelial PHD inactivation, we performed flow cytometric analysis (Supplemental Figure 10). Because at day 14 after uIRI, there was an established maladaptive repair phenotype, we focused our analysis on day 8, an earlier time point that may reveal changes in immune responses contributing to the observed phenotype. Notably, day 8 postischemic kidneys of *PHD^TiEC^* showed a 2.9-fold increase in macrophages (CD45^+^CD11b^+^F4/80^+^) (Figure 6, C and D) compared with controls. F4/80 immunostaining showed diffuse and variable distribution within the postischemic kidneys of *PHD^TiEC^* mutants (Figure 6D). Taken together, these data support that endothelial postischemic PHD inactivation promotes maladaptive inflammatory responses and increased macrophage infiltration.

To investigate how mRECs interact with macrophages, we employed NicheNet, a previously published algorithm, which utilizes expression data to infer the potential impact of ligand-receptor interactions on specific targets by integrating existing knowledge of signaling and regulatory networks (35). We assigned mRECs as “senders” and the different macrophage clusters as “receivers.” NicheNet analysis showed that ligands expressed by mRECs could explain 18 out of the 167 DEGs found in the Mφ1 cluster of *PHD^TiEC^* mutant compared with control, while the impact was more limited for the other macrophage clusters (2/64 for Mφ2; 5/32 for Mφ3; Mφ4 and pMφ showed no interactions) (Figure 6E). Several notable ligands and target genes emerged from analyzing the interaction of mRECs with Mφ1 cluster. One prominent example is *Cxcl12*, which exhibited high expression in mRECs of *PHD^TiEC^* mutant. CXCL12 is a known HIF target gene that can interact with the G protein–coupled chemokine receptor CXCR4, which is abundantly expressed in macrophages. This interaction has the potential to modulate macrophage activation, contributing to profibrotic function (36). Pathway analysis by GSEA identified several Hallmark pathways substantially enriched in the Mφ1 cluster of *PHD^TiEC^* mutant. These pathways included hypoxia, inflammatory response, TNFA signaling via NF-κB, mTORC1 signaling, complement, and unfolded protein response (Figure 6F and Supplemental Data File 5). Taken together, these results suggest that postischemic endothelial PHD inactivation triggers an endothelial driven proinflammatory milieu with macrophages, which may contribute to tissue injury and fibrosis following kidney IRI.

### Postischemic inactivation of endothelial HIF signaling inhibits kidney fibrosis following kidney IRI.

**Figure 7:**
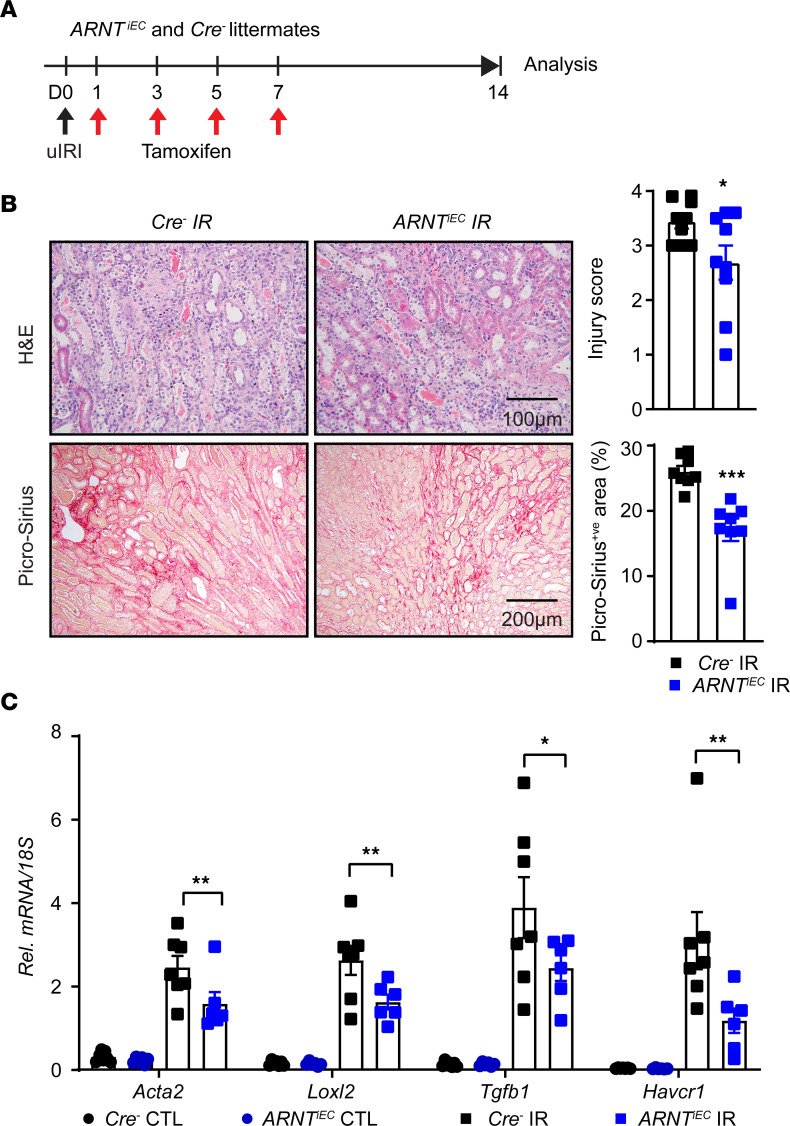
Postischemic inactivation of endothelial ARNT promotes adaptive repair following postischemic kidney injury. (**A**) Experimental schematic illustrates the timing of unilateral renal artery clamping, tamoxifen administration, and analysis. (**B**) Representative images of H&E- and Picrosirius red–stained sections from day 14 postischemic kidneys of *ARNT^iEC^* mutants and their *Cre^–^* littermates. Right panels show tubular injury score (top) and semiquantitative analysis of Picrosirius red^+ve^ area in the indicated genotypes. Scale bars: 100 μm (H&E); 200 μm (Picrosirius red). (**C**) mRNA levels of *Acta2*, *Loxl2*, *Tgfb1*, and *Havcr1* in IR and CTL kidneys from *ARNT^iEC^* mice and their *Cre^–^* controls at day 14 after uIRI. All bars show mean ± SEM. For **B**, unpaired *t* test with Welch’s correction was used. For **C**, statistics were determined using 1-way ANOVA with Šidák’s correction for multiple comparisons. *n* = 6–9. *P < 0.05; **P < 0.01; ***P < 0.001.

We next asked whether activation of endothelial HIF signaling in the context of PHD inactivation is a driver of the observed maladaptive repair phenotype. Therefore, we generated mice that allow EC-inducible inactivation of Aryl hydrocarbon receptor nuclear translocator (ARNT), the constitutive HIF-1β subunit required for activation of HIF signaling by crossing *Cdh5(PAC)CreER^T2^* transgenics with *Arnt* floxed mice. The resulting *Cdh5(PAC)CreERT2;* *ARNT^fl/fl^* (*ARNT^iEC^*) and *Cre^–^* littermates were subjected to uIRI followed by tamoxifen treatment and analysis on day 14 as indicated in Figure 7A. Notably, postischemic endothelial ARNT inactivation significantly ameliorated kidney injury and fibrosis assessed by H&E and Picrosirius red staining (Figure 7B). Furthermore, mRNA levels of *Acta2*, *Loxl2*, *Tgfb1*, and hepatitis A virus cellular rzeceptor 1 (*Havcr1*) were also significantly reduced in day 14 post-IR kidneys of *ARNT^iEC^* mice compared with *Cre^–^* littermates (Figure 7C). In summary, these results show that postischemic inactivation of endothelial HIF signaling is sufficient to suppress AKI to CKD transition following ischemic kidney injury.

### Treatment with the MCT4 inhibitor syrosingopine restores adaptive repair in postischemic kidney injury of PHD^TiEC^ mice.

**Figure 8:**
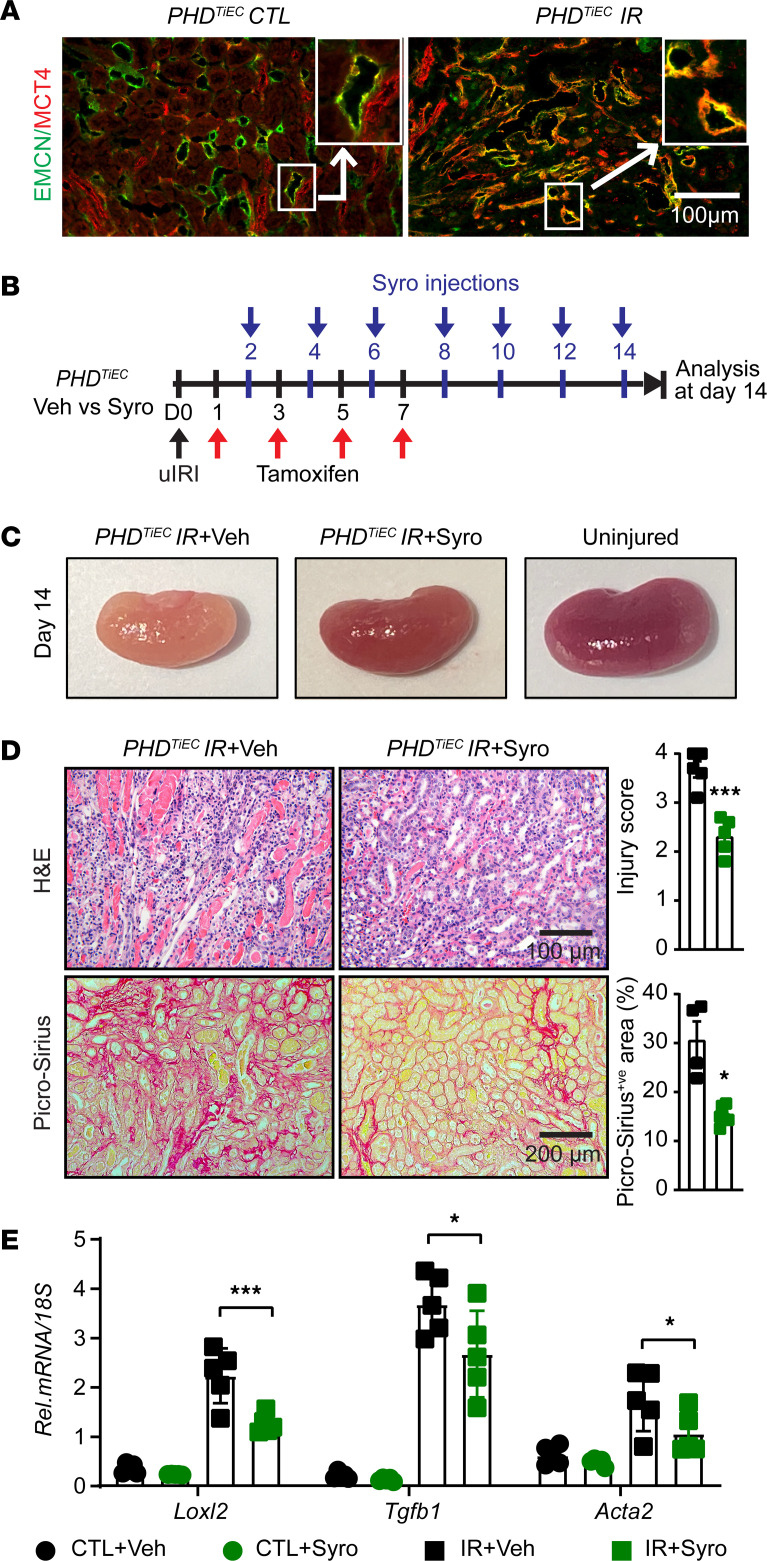
Post-IRI treatment with the MCT4 inhibitor syrosingopine restores adaptive kidney repair in *PHD^TiEC^* mice. (**A**) Representative images of immunofluorescence staining for MCT4 (red) and EMCN (green) of contralateral and day 14 postischemic kidneys from *PHD^TiEC^* mice. Zoom-in panels show the increased expression of MCT4 in EMCN^+ve^ cells in *PHD^TiEC^* postischemic kidney. Images were captured using a Nikon Ti2 Widefield fluorescence microscope. Scale bar: 100 μm. (**B**) Schematic illustrates the timing of unilateral renal artery clamping, tamoxifen administration, treatment with syrosingopine, and analysis at day 14 after uIRI. (**C**) Representative images of uninjured kidney compared with day 14 postischemic kidneys treated with vehicle or syrosingopine. All mice are *PHD^TiEC^* mutants. (**D**) Representative images of H&E- and Picrosirius red–stained day 14 postischemic kidneys from vehicle- versus syrosingopine-treated *PHD^TiEC^* mutants. Right: tubular injury score and semiquantitative analysis of Picrosirius red^+ve^ area of day 14 postischemic kidneys for the indicated experimental groups. Scale bars: 100 μm (H&E); 200 μm (Picrosirius red). (**E**) mRNA levels of *Loxl2*, *Tgfb1*, and *Acta2* in CTL and IR kidneys from vehicle- or syrosyngopine-treated *PHD^TiEC^* mice on day 14 after uIRI. Data are represented as mean ± SEM. For **D**, statistics were determined by unpaired *t* test with Welch’s correction. For **E**, statistics were determined using 1-way ANOVA with Šidák’s correction for multiple comparisons. *n* = 4–6. ^*^P < 0.05; ^***^P < 0.001. veh, vehicle; Syro, syrosingopine.

As the primary metabolic response of the postischemic endothelium to HIF stabilization in the context of PHD inactivation was increased glycolysis, we next focused on this aspect. While increased lactate generation and efflux via the transporter MCT4 represent the final steps of hypoxia-induced glycolysis (37), lactate export was recently shown to be among the 4 key steps controlling glycolytic flux (38). *Slc16a3*, the gene encoding MCT4, was markedly increased in mRECs of postischemic *PHD^TiEC^* kidney as well as in ECs of human kidneys with severe AKI. Indeed, immunofluorescence staining confirmed high expression of MCT4 protein in the postischemic kidney endothelium of *PHD^TiEC^* (Figure 8A and Supplemental Figure 11). We next sought to investigate whether increased endothelial MCT4 causally contributes to the maladaptive postischemic kidney repair observed in the *PHD^TiEC^* mice. To this end, we used syrosingopine, a dual inhibitor of monocarboxylate transporters MCT1 and MCT4 (60-fold higher potency on MCT4), which leads to end-product inhibition of glycolysis (39). As depicted in Figure 8B, *PHD^TiEC^* mice were treated with syrosingopine or vehicle starting on day 2 after uIRI and then every other day until day 14 after uIRI. Strikingly, treatment with syrosingopine significantly suppressed tubular injury and fibrosis as indicated by approximately 37% and approximately 51% reduction in injury scoring and collagen accumulation, respectively, compared with vehicle (Figure 8, C and D). Furthermore, the mRNA levels of profibrotic genes *Loxl2*, *Tgfb1*, and *Acta2* were significantly downregulated in the syrosingopine-treated group compared with the vehicle-treated group (Figure 8E). Because syrosingopine could also exert its effects through MCT1 inhibition, *PHD^TiEC^* mice were treated with the MCT1 inhibitor AZD 3965. Nevertheless, MCT1 inhibition alone had no effect on tubular injury and fibrosis on 14 after uIRI (Supplemental Figure 12). Taken together, our studies demonstrate that inhibition of endothelial MCT4 by syrosingopine restores adaptive kidney repair following IRI in *PHD^TiEC^* mutants.

### MCT4 inhibition diminishes endothelial-derived proinflammatory responses induced by hypoxia reoxygenation and IL-1β.

**Figure 9:**
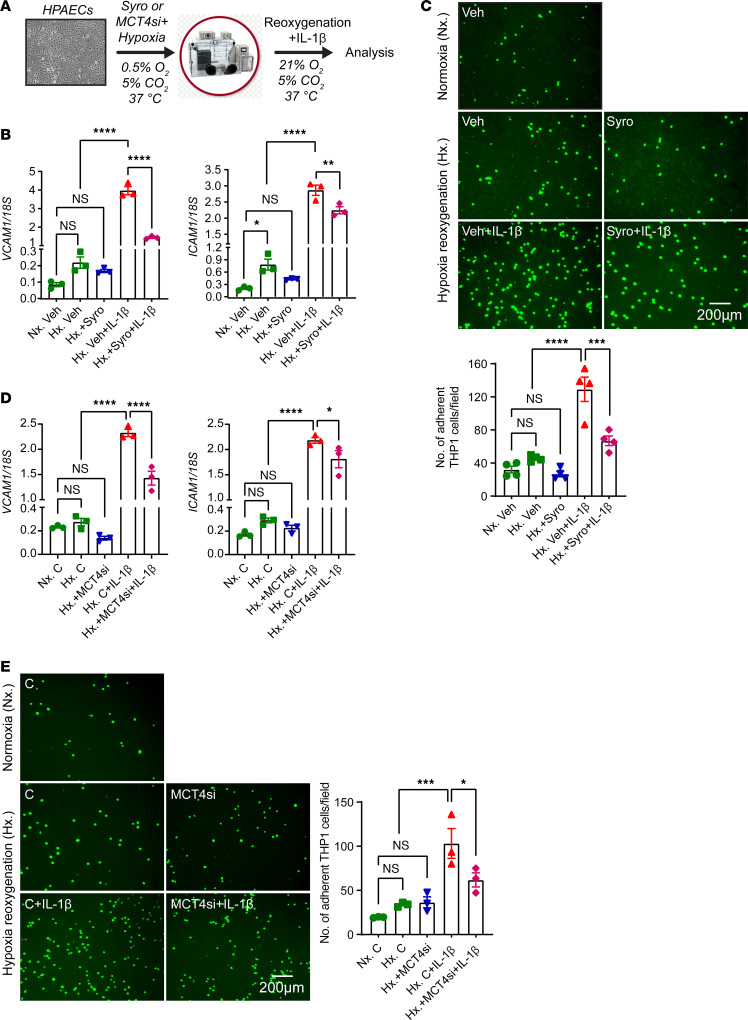
Syrosingopine or MCT4 knockdown suppresses the expression of EC adhesion molecules in HPAECs activated by hypoxia/reoxygenation and IL-1β. (**A**) Experimental schematic for HPAECs subjected to 0.5% O_2_ for 18 hours in the presence of syrosingopine (5 μM) or MCT4 siRNA followed by reoxygenation for 8 hours in the presence of IL-1β (1 ng/ml). (**B**) mRNA levels of *VCAM1* and *ICAM1* in syrosingopine- vs vehicle-treated HPAECs, that were activated by hypoxia/reoxygenation and IL-1β. (**C**) THP1 monocyte adhesion to inflamed ECs. THP1 monocyte cells, labeled with green CMFDA dye, were introduced on a monolayer of HPAECs that had been subjected to the indicated experimental conditions. Following a 90-minute incubation period, floating cells were washed away and adhered THP1 cells were visualized using a fluorescent microscope and subsequently quantified. Representative images of fluorescent THP1 cells attached to ECs in different experimental groups are presented. Scale bar: 200 μm. (**D**) mRNA expression of *VCAM1* and *ICAM1* in HPAECs transfected with control or MCT4 siRNA and subjected to hypoxia/reoxygenation and IL-1β. (**E**) THP1 monocyte adhesion to inflamed ECs under the same experimental conditions as in **D**. Scale bar: 200 μm. Data are represented as mean ± SEM. Statistics were determined by 1-way ANOVA with Šidák’s correction for multiple comparisons. *n* = 3–4. ^*^P < 0.05; ^**^P <0.01; ^***^P <0.001; ^****^P < 0.0001. Nx, normoxia; Hx, hypoxia/reoxygenation; C, negative control siRNA; MCT4si, MCT4siRNA.

To assess whether MCT4 inhibition acts cell autonomously in ECs to suppress proinflammatory responses, we performed in vitro experiments in which human primary pulmonary artery endothelial cells (HPAECs) were activated by 18 hours exposure to hypoxia (0.5% O_2_), followed by reoxygenation for 8 hours in the presence of IL-1β treatment (1 ng/mL) (Figure 9A). To inhibit MCT4, we first used syrosingopine at a concentration of 5 μM, which diminished lactate secretion, as indicated by reduction in extracellular lactate levels (Supplemental Figure 13A). We found that syrosingopine significantly suppressed the hypoxia-reoxygenation and IL-1β–induced upregulation of *VCAM1* and *ICAM1* (Figure 9B) compared with controls leading to an approximately 48% reduction in adhesion of monocytes (THP-1 cells) to HPAECs (Figure 9C). Mechanistically, this response could be due to suppression of NF-κΒ activity by syrosingopine as indicated by reduced phospho-p65 protein levels (Supplemental Figure 14). Consistent responses were observed when we used siRNA against MCT4. With an approximately 67% knockdown efficiency (Supplemental Figure 13B), *MCT4siRNA* significantly suppressed *VCAM1* and *ICAM1* transcripts in ECs stimulated by hypoxia-reoxygenation and IL-1β compared with negative control siRNA, reducing adhesion of monocytes to HPAECs by approximately 40%. (Figure 9, D and E). Because syrosingopine could act by inhibiting MCT1, we also conducted experiments with the MCT1 inhibitor AZD 3965. Nevertheless, AZD 3965 had no effect on the transcriptional induction of *VCAM1* and *ICAM1* in HPAECs stimulated by hypoxia-reoxygenation and IL-1β (Supplemental Figure 15). Taken together, these results demonstrate that MCT4 inhibition suppresses EC interaction with monocytes in a cell-autonomous manner, an effect that may contribute to favorable effects exerted by syrosingopine, when given after kidney IRI.

## Discussion

In this study, we show that the regulation of endothelial oxygen sensing signaling has critical implications in postischemic kidney repair. Within the kidney endothelium, we demonstrate the expression of PHD1 and PHD3 isoforms along with PHD2 and show their critical contribution in regulating HIF signaling in the renal vascular bed. By investigating the outcomes of kidney repair in different conditional knockout strains, we provide evidence that activation of HIF signaling through concurrent loss of the 3 PHD isoforms and not PHD2 alone dictates postischemic kidney repair. We furthermore identify the endothelial glycolysis/MCT4 axis as a promising therapeutic target. Pharmacological inhibition of MCT4 holds the potential to interrupt the transition from AKI to CKD by mitigating inflammation.

PHD enzymes are nonheme iron-dependent dioxygenases that catalyze the 4-electron reduction of O_2_. Specifically, they incorporate each O_2_ atom, respectively, into the tricarboxylic acid (TCA) cycle metabolite 2-oxoglutarate (2OG) and the substrate polypeptide, forming succinate, carbon dioxide, and *trans*-4-hydroxylated prolyl products (10). With *K*m values falling within the 230–250 μM range, PHDs are sensitive to fluctuations in local O_2_ in tissues, thus functioning as oxygen sensors (10). While PHD2 is considered the main oxygen sensor (10, 40), the other isoforms have been implicated in regulating hypoxia signaling (13). Consistently, we have demonstrated an overlapping pattern of expression for PHDs in kidney endothelium, which probably allows notable compensatory responses. This may explain the lack of HIF stabilization when endothelial PHD2 was individually deleted (17) in contrast to the robust HIF activation we observed when the 3 PHD isoforms were concurrently inactivated. While the regulation of HIF by PHDs in different vascular beds requires further investigation, our findings are particularly relevant for IRI, where severe hypoxia could limit the activity of all PHD isoforms. Furthermore, because PHDs are regulated not only by oxygen but also by factors such as 2OG, oxidative stress, and abnormal concentrations of endogenous metabolites, PHD-mediated hydroxylation can be altered even under normoxia, creating a “pseudo-hypoxic state” (10). For example, succinate, a TCA cycle metabolite known to accumulate with IRI, can inhibit all 3 PHDs (41, 42). Therefore, various hypoxic and pseudohypoxic signals within the postischemic kidney microenvironment can alter the activity of PHDs dictating tissue remodeling.

Our findings are clinically relevant, as pan-PHD inhibitors are already approved for treating renal anemia. While no serious adverse effects have been widely reported, the FDA analyses of the ASCEND-ND (ClinicalTrials.gov NCT02876835) trial suggested a potential increased risk of AKI with daprodustat, a HIF prolyl hydroxylase inhibitor, compared with the conventional erythropoiesis-stimulating agent darbepoetin alfa (43, 44). Given the key role of PHD2 in renal erythropoietin synthesis (45), PHD2-selective inhibitors would likely offer a safer alternative, although none are currently available. Therefore, further research is required to define the specific functions of PHD isoforms in different cell types optimizing both the efficacy and safety of oxygen-sensing based therapies.

Capillary rarefaction was exacerbated after ischemic kidney injury in the setting of postischemic endothelial PHD inactivation and was associated with the presence of partially “dedifferentiated” ECs, as reflected by the EC-EndMT cluster revealed by scRNA-Seq. EndMT could be induced directly by HIF activation in the context of endothelial PHD inactivation, as indicated by previous studies linking HIFs to EndMT in pulmonary and cardiac fibrosis by upregulating the snail family transcriptional repressor 1 (SNAI1) (46, 47). While the contribution of EndMT in our model is unclear, impaired angiogenic capacity could represent another EC autonomous mechanism by which PHD inactivation leads to capillary drop-out, as we and others recently showed in cultured ECs (48, 49). Regardless of the underlying mechanism, by inducing kidney tissue hypoxia, capillary rarefaction contributes to a vicious cycle of maladaptive responses (50). For example, Lovisa et al. showed an intriguing link between endothelial dysfunction and impaired tubular epithelial metabolism shifting from fatty acid oxidation to glycolysis (51).

Postischemic endothelial PHD inactivation augmented interstitial fibrosis and inflammation following kidney IRI, and these responses were associated with robust HIF activation. Based on our previous studies in which constitutive deletion of endothelial HIF-2 enhanced IRI-induced kidney fibrosis and inflammation (18), and preischemic inactivation of endothelial PHD2 was protective (17), our findings were unanticipated and raise important questions. Does the timing of endothelial PHD inactivation in the setting of established kidney injury dictate the repair outcomes, does the nonphysiologic endothelial HIF activation drive these unfavorable responses, or are HIF-independent effects of PHDs involved? These possibilities are not mutually exclusive. Pharmacological approaches of PHD inhibition applied prior to ischemic kidney injury have provided renoprotective effects, which though were not observed when HIF signaling was activated during the postischemic phase (52). By employing a genetic approach of inducible EC-specific targeting, our studies identify temporal context as a critical factor in determining kidney injury responses to activation or inhibition of the HIF/PHD pathway. Importantly, our system is not confounded by the potential off-target effects of PHD inhibitors on other 2OG-dependent dioxygenases. Furthermore, the favorable effects observed in the context of postischemic endothelial ARNT deletion support a maladaptive role for endothelial HIF signaling in reparative processes after kidney injury. Nevertheless, non-HIF hydroxylation substrates of PHD could also contribute to the observed outcomes by PHD inhibition. For example, PHD inhibition could reduce the hydroxylation of IKKβ derepressing NF-κΒ signaling (53, 54), a response known to induce endothelial adhesion molecules binding and transmigration of leukocytes.

ECs are known to use glycolysis to meet more than 80% of their adenosine triphosphate (ATP) demands (55, 56), preserving oxygen for perivascular cells. Our study now unveiled that glycolysis can be further enhanced in postischemic RECs of *PHD^TiEC^* mice, which is in agreement with the metabolomic changes we recently reported in ECs treated with the pan-PHD inhibitor dimethyloxalylglycine (DMOG) (49). Importantly, increased endothelial glycolysis was associated with proinflammatory changes that lead to enhanced macrophage infiltration. In accordance with our findings, recent studies reported induction of glycolysis in ECs subjected to the inflammatory mediators TNFA and lipopolysaccharide (LPS) (37, 57), both known to induce HIF activation (58, 59). In this setting, among the upregulated glycolytic genes, PFKFB3 was identified as a key regulator of this metabolic reprogramming. Notably, genetic or pharmacologic inactivation of PFKFB3 abrogated inflammation in various disease models such as and LPS and pulmonary hypertension, linking PFKFB3-driven glycolysis to endothelial proinflammatory pathways (57, 60). Importantly, our analysis of scRNA-Seq data from kidneys of patients with severe AKI demonstrates increased endothelial glycolysis and activation of hypoxia and inflammatory pathways, highlighting a role for PHD/HIF in regulating the immunometabolic response of kidney endothelium.

The HIF-1 induction of MCT4 allows the efficient export of lactic acid and enables glycolytic tumors to grow within an acidic microenvironment (61, 62). Furthermore, because MCT4-mediated lactic acid transport can regulate glycolytic flux, blocking MCT4 has been proposed as a strategy to suppress tumor growth in different cancers by disrupting the Warburg effect (63). Given that (a) ECs resemble cancer cells in their preferential use of glycolysis and (b) MCT4 was markedly upregulated by endothelial PHD inactivation in the postischemic kidney, we postulated that MCT4 inhibition might promote adaptive kidney repair by suppressing endothelial glycolysis. To test this hypothesis, we treated *PHD^TiEC^* mice with syrosingopine starting on day 2 after uIRI and found that MCT4 inhibition remarkably reduced the tubular injury and development of fibrosis at day 14 after uIRI. To assess whether MCT4 inhibition had EC autonomous effects, HPAECs activated to a proinflammatory phenotype by hypoxia-reoxygenation and IL-1β were treated with syrosyngopine or siMCT4. Both genetic silencing and pharmacologic MCT4 inhibition suppressed the expression of EC-adhesion molecules, reducing the adhesion of THP1 cells. Interestingly, inhibition of CD147, a broadly expressed transmembrane glycoprotein that is essential for MCT4 function (64), has been reported to reduce postischemic brain inflammatory cell infiltration by reduced NF-κB activation in brain microvascular ECs (65). While this mechanism could explain our findings, a broader reprogramming of EC metabolism by MCT4 inhibition may be a key driver. For instance, Cluntun et al. showed that MCT4 inhibition shifted glucose flux from lactate to citrate and the TCA cycle in cardiomyocytes (66). Further studies are needed to investigate how metabolic reprogramming by MCT4 inhibition is linked to endothelial function.

In summary, our genetic studies demonstrate that postischemic endothelial PHD inactivation leads to HIF activation and maladaptive kidney repair, which can be reversed by MCT4 inhibition. Our findings merit further consideration of targeting the kidney endothelial hypoxia-driven glycolysis/MCT4 axis as a therapeutic strategy to halt the AKI to CKD transition.

## Methods

### Sex as a biological variable

Samples from both sexes were included in the analysis of human data. Animal studies were conducted on male mice except for an IRI experiment on female *PHD^TiEC^* mice showing variable outcomes. Whether the reported findings in male mice are relevant to females requires future investigation.

### Generation of mice, genotyping, and animal procedures

The generation and genotyping of *Phd1* (*Egln2*), *Phd2* (*Egln1*), *Phd3* (*Egln3*), and *Arnt* (*Arnt*) floxed mice have been described previously (45, 67, 68). For inducible endothelial-specific inactivation of the floxed alleles, we used the *Cdh5(PAC)-CreER^T2^* transgenic mouse line, provided by Ralph Adams (Max Planck Institute for Molecular Biomedicine, Department of Tissue Morphogenesis, Münster, Germany) (20). To examine recombination efficiency in the postischemic kidney, we generated *Cdh5(PAC)CreER^T2^;mT/mG* mice by crossing the ROSA26-ACTB-tdTomato,-EGFP reporter mice (JAX stock number 007576) to the *Cdh5(PAC)CreER^T2^* transgenic mouse line. Cre-mediated inactivation was induced by 4 i.p. injections of tamoxifen dissolved in 10% ethanol in corn oil at 20 mg/mL (3 mg/mouse) given every other day. Mice were maintained in a specific pathogen–free facility on a 12-hour light/12-hour dark cycle. Littermates were randomly assigned to experimental groups.

Mice 8–10 weeks of age underwent renal IRI surgery as previously described (17). Anesthesia was induced by i.p. injection of xylazine (10 mg/kg) and ketamine (90-120 mg/kg). After making a small midline abdominal incision, the left renal pedicle was occluded by applying a microaneurysm clamp for 25 minutes in males and 30 minutes in females, while the right kidney was kept intact as an internal control in the uIRI. For bIRI, clamps were applied in both renal pedicles for 23 minutes. After the specified time, the clamps were removed, and reperfusion was visually confirmed. The incision site was closed with a 6-0 suture followed by the closure of the skin wound with Michel miniature clips. Throughout the surgical procedure, body temperature was monitored by a rectal probe and controlled with a heating pad at 37°C.

For in vivo MCT4 inhibition studies, mice were treated with syrosingopine (7.5 mg/kg) i.p. or vehicle starting on day 2 after IRI every other day until dissection. For MCT1 inhibition studies, mice were treated with AZD 3965 (30 mg/kg) by gavage.

### DNA, RNA, and protein analyses

DNA and RNA were extracted and used for genomic or real-time PCR analysis, as previously described (14). Mouse and human primer sequences are listed in Supplemental Table 3. Real-time PCR was run in the QuantStudio 3 Real-Time PCR system (Applied Biosystems) using SYBR green or TaqMan PCR Master Mix. 18S rRNA was used for normalization.

Nuclear protein was extracted using NE-PER Nuclear and Cytoplasmic Extraction Reagents (Thermo Fisher Scientific) following the provided instructions. The extracted nuclear protein samples were then subjected to separation on an SDS-PAGE gel, transferred on a nitrocellulose membrane, and subsequently incubated with HIF-1α antibodies (Cayman; catalog 10006421), HIF-2α antibodies (Novus Biologicals; catalog NB100-122), NF-κB p65 (Cell Signaling Technology; catalog 8242S), or phospho–NF-κB p65 (Ser536) (Cell Signaling Technology; catalog 3033S) at 4°C. Following an overnight incubation, the nitrocellulose membrane was washed and incubated with a secondary antibody (Novus Biologicals) for 90 minutes at 4°C. Chemiluminescent signal detection was performed using SuperSignal West Femto Chemiluminescent Substrate (Thermo Fisher Scientific), and membranes were imaged on the iBright Imaging System (Thermo Fisher Scientific).

### Histopathological and immunofluorescence analysis

For the histological examination, kidneys were fixed in 10% formalin buffer and paraffin blocks were prepared. To assess tubular damage and interstitial fibrosis, 5 μm transverse plane kidney sections were separately stained with H&E, Masson’s trichrome, and Picrosirius red. Tubular injury was semi-quantitatively scored by determining the percentage of tubules in the corticomedullary junction that displayed necrosis, loss of brush border, cast formation, and tubular dilatation (0, unaffected; 1, 1%–25%; 2, 26%–50%; 3, 51%–75%; 4, 76%–100%) (69). Fibrosis was assessed by the percentage of Masson’s trichrome^+ve^ and Picrosirius red^+ve^ area determined with ImageJ software (http://rsbweb.nih.gov/ij). For tubular injury scoring as at least 10 random visual fields of corticomedullary region were captured and analyzed per kidney section for each sample at a magnification of ×200. For quantification of fibrosis by Masson’s trichrome^+ve^ and Picrosirius red^+ve^ area at least 5 random visual fields of cortex and medulla were captured and analyzed per kidney section for each sample at a magnification of ×200.

Immunofluorescence staining was performed using primary antibodies against PHD1 (Abcam; catalog ab113077), PHD2 (Novus Biologicals; catalog NB100-137), PHD3 (Novus Biologicals; catalog NB100-139), endomucin (Abcam; catalog ab106100), F4/80 (Abcam; catalog ab6640), MCT4 (Proteintech; 22787-1-AP), CD31 (R&D Systems; catalog AF3628), ICAM1 (Abcam, catalog ab119871), aSMA (Abcam; catalog ab7817) and CXCL12 (Invitrogen; catalog PA5-89116). Goat anti-rat Alexa Fluor 488 (Invitrogen; catalog A11006), donkey anti-goat Alexa Fluor 488 (Invitrogen; catalog A11055), goat anti-rabbit Alexa Fluor 594 (Invitrogen; catalog A32740), goat anti-mouse Alexa Fluor 594 (Invitrogen; catalog A11032), and goat anti-rabbit Alexa Fluor 647 (Invitrogen, catalog A32733) were used as secondary antibodies. VECTASHIELD Vibrance Antifade Mounting Medium with DAPI (Vector Laboratories Inc., catalog NC1601055) was used as mounting media. Images were captured by a fluorescence microscope (Nikon Ti2 widefield microscope) and analyzed using Fiji software (ImageJ). Percentage of endomucin^+ve^ area was determined with ImageJ software.

### BUN and GFR measurements

Serum BUN levels were measured using the QuantiChrom Urea Assay Kit (BioAssay Systems) following the manufacturer’s instructions. Transcutaneous GFR was measured via the transcutaneous clearance of FITC-Sinistrin using a miniaturized fluorescence detector (NIC-Kidney device) (70).

### Preparation of single-cell suspension

Injured kidneys from *PHD^TiEC^* and *Cre^–^* mice were collected on day 14 after uIRI. Single-cell suspensions were prepared using the Multi Tissue Dissociation Kit 2 (Miltenyi Biotec; catalog 130-110-203) following the instructions provided with the kit. Briefly, IR kidneys were cut into 6–8 pieces using single edge blades and placed in gentleMACS C tube with 5 ml dissociation buffer (4.8 mL of buffer X, 50 μL of enzyme P, 50 μL of buffer Y, 100 μL of enzyme D, and 20 μL of enzyme A provided in Multi Tissue Dissociation Kit 2). gentleMACS C tubes were placed in a gentleMACS Dissociator (Miltenyi Biotec) and kidneys were dissociated using program Multi_E_01. Dissociated kidneys were incubated for 30 minutes at 37°C with continuous rotation using the MACSmix Tube Rotator (Miltenyi Biotec). gentleMACS C tubes were then placed in the gentleMACS Dissociator and the Multi_E_02 program was run. gentleMACS C tubes were detached, and 10 mL neutralizing buffer (1× PBS with 2% fetal bovine serum) was added to stop the dissociation reaction. Cell suspension was filtered through 100 μm and then 30 μm cell strainer. Cell suspension was centrifuged at 400*g* for 10 minutes. The cell pellet was incubated with 1 mL of RBC lysis buffer on ice for 1 minute and after the addition of 10 mL neutralizing buffer, cells were recentrifuged at 400*g* for 5 minutes. Cells were suspended in 10 mL neutralizing buffer and centrifuged again at 100*g* for 5 minutes. Finally, the cell pellet was suspended in 1× PBS with 0.04% BSA and filtered through 30 μm cell filter. Cell number and viability were analyzed using the Nexcelom Cellometer Auto 2000 with the AOPI fluorescent staining method. This method resulted in single-cell suspensions with more than 80% viability.

### scRNA-Seq library generation and sequencing

Sixteen thousand cells were loaded into the Chromium Controller (10X Genomics; catalog PN-120223) on a Chromium Next GEM Chip G (10X Genomics, catalog PN-1000120) and processed to generate single-cell gel beads in the emulsion (GEM) according to the manufacturer’s protocol. The cDNA and library were generated using the Chromium Next GEM Single Cell 3′ Reagent Kit, version 3.1 (10X Genomics; catalog PN-1000286) and single Index Kit T Set A (10X Genomics; catalog PN-1000213) according to the manufacturer’s manual. For the cell hashing library, it was constructed according to BioLegend’s published protocol on HTO library preparation guidelines with single index adapters. Quality control for the constructed library was performed by the Agilent Bioanalyzer High Sensitivity DNA Kit (Agilent Technologies; catalog 5067-4626), while the Qubit DNA HS Assay Kit was used for qualitative and quantitative analysis. The multiplexed libraries were pooled and sequenced on an Illumina HiSeq 4000 sequencer with 2X50 paired-end kits using the following read lengths: 28 bp for read1 for cell barcode and UMI, and 90 bp for read2 for transcript. Raw sequencing data in base call format (.bcl) was demultiplexed using Cell Ranger from 10X Genomics, converting the raw data into FASTQ format. Cell Ranger was also used for alignment of the FASTQ files to the mouse reference genome (mm10) and to count the number of reads from each cell that align to each gene.

### scRNA-Seq data analysis

The resulting matrix files, which summarize the alignment results, were imported into Seurat (Satija Lab, NYGC) for further analysis. In Seurat (version 4.3.0), each individual sample was transformed to Seurat object. Only genes expressed in 3 or more cells and cells expressing at least 200 genes or more were used for further analysis. Cells with fewer than 500 unique molecular identifier (UMI) counts (cell fragments) and more than 100,000 UMI counts (potentially cell duplets) were excluded (71). Furthermore, cells with mitochondrial gene percentage over 50% and low complexity cells such as red blood cells with less than 0.8 log_10_ genes per UMI counts were also excluded from the analysis (71). Merged data were normalized and scaled, and PCA was done. The top 20 principal components were chosen for neighbors and cell clustering was performed with a resolution of 0.8. The cell clusters were visualized in 2-dimensional space by UMAP. Both genotypes showed similar clustering and cell populations in separate and overlapping DimPlot (72). To identify cell clusters, marker genes were assessed using the “FindAllmarkers” function of Seurat with the setting of min.pct of 0.25 and logfc.threshold of 0.25. Based on marker analysis, one cluster predominately enriched with lncRNAs Gm26917 and Gm42418 associated with ribosomal RNA contamination was excluded (73). The remaining 28 clusters were annotated based on the expression of top marker genes supported by published studies (Supplemental Table 1). Differential gene expression analysis was performed, and genes with adjusted *P* < 0.05, log_2_FC > 0.2 were considered significantly regulated. GSEA was performed using GSEA software (version 4.2.3) and by EnrichR (https://maayanlab.cloud/Enrichr/).

### Public scRNA-Seq data analysis

#### Murine scRNA-Seq data.

Adult mouse kidney endothelial scRNA-Seq data were extracted from the EC Atlas database created by the Carmeliet group (21). Data were processed and analyzed using standard Seurat’s workflow for data processing and normalization (https://satijalab.org/seurat/articles/pbmc3k_tutorial.html). Cell clusters were generated with 0.7 resolution and identified by analyzing the expression of EC marker genes (*Plvap*, *Igfbp3*, *S100a6*, *Sox17*, *Igfbp7*, *Aqp1*, *Pi16*, *Plat*, *Ehd3*, *Cyp4b*, and *Lpl*) (74).

#### Human scRNA-Seq data.

The expression of EGLNs genes in human kidney ECs was assessed by extracting human kidney biopsy scRNA-Seq datasets generated by KPMP and available via the NCBI’s Gene Expression Omnibus (GEO GSE140989; Supplemental Table 1) (22). Seurat files were created and combined following Seurat’s standard workflow. The EC cell cluster was identified based on the expression of endothelial markers EMCN, CD34, PLVAP, and EHD3. After extraction, ECs were further clustered into 3 EC subclusters, which were identified based on the expression of different EC markers (*PLVAP*, *RGCC*, *MARCKS*, *SPARCL1*, *IGFBP7*, *TGFBR2*, *SOST*, *PLAT*, *EHD3*, *MEG3*, *SERPINE2*, *KCTD12*, *LTBP4*, *CLDN5*, and *IGF2*) (22).

#### Human snRNA-Seq data.

Human snRNA-Seq datasets from AKI and control kidney tissues (8 AKI; 6 controls) were extracted from Gene Expression Omnibus (GSE210622) (34). Raw data were analyzed using Seurat’s best practice workflow for data integration using the reciprocal PCA approach (https://satijalab.org/seurat/articles/integration_rpca.html) with default parameters. Clusters were then analyzed for marker genes, and EC clusters (*CD34*^+^ and *EMCN^+^*) were extracted, combined, and analyzed following Seurat’s best practice standard workflow for data integration and analysis (https://satijalab.org/seurat/articles/integration_introduction.html). Differential gene expression analysis was performed, and significantly upregulated genes (adjusted *P* < 0.01, log_2_FC > 0.25) were subjected to Hallmark analysis by EnrichR (https://maayanlab.cloud/Enrichr/). The expression levels of glycolytic genes in AKI and control kidney ECs were directly derived from the publicly accessible online interface (https://shiny.mdc-berlin.de/humAKI/) (34).

### Flow cytometry

Following perfusion with PBS, kidneys were harvested, minced, and incubated in dissociation solution (Multi Tissue Dissociation Kit 2; Miltenyi Biotec; catalog 130-110-203) for 30 minutes at 37ºC. After dissociation, cells were passed through a 70 and 40 μm filter, and 10 mL of cold staining buffer (PBS + 2% fetal bovine serum) was added. Cells were centrifuged (400*g* for 10 minutes), and cell pellets were resuspended in cold RBC lysis buffer, centrifuged, and resuspended in staining buffer. Following incubation with CD16/32 (Fc block), single-cell suspensions were labeled with different fluorophore-tagged antibodies. The following antibodies were used: CD16/32 (eBioscience, catalog 14-0161-85), CD45 (clone 30-F1, BioLegend; catalog 103140), F4/80 (clone BM8, BioLegend; catalog 123110), CD11b (clone M1/70, BioLegend; catalog 101216), CD3 (clone 17A2, BioLegend; catalog 100210), Ly6C (clone HK1.4; BioLegend; catalog 128010), Ly6G (BD Biosciences; catalog 560600), and CD31 (clone 390, Invitrogen; catalog 17-0311-82).

### Cell culture

HPAECs were obtained from ATCC and grown on gelatin-coated dishes in Endothelial Cell Basal Medium-2 (Lonza, catalog CC-3156) supplemented with EGM-2 SingleQuots Supplements (Lonza, catalog CC-4176). MCT4 siRNA was purchased from QIAGEN (FlexiTube GeneSolution GS9123 for SLC16A3; catalog 1027416). AllStars negative control siRNA (QIAGEN) was used as control siRNA. HiPerFect transfection reagent (QIAGEN, catalog 301707) was used for siRNA transfection experiments. For hypoxia experiments, MCT4siRNA transfected and syrosingopine or AZD 3965-treated HPAECs and their corresponding controls (AllStars negative control transfected and vehicle-treated, respectively) were incubated for 18 hours in a hypoxia chamber (Coy Laboratory Products) at 0.5% O_2_, 37˚C in a 5% CO_2_ humidified environment. During reoxygenation, HPAECs were treated with 1 ng/ml IL-1β (MilliporeSigma, catalog H6291) for 8 hours.

#### Endothelial-monocyte cell adhesion assay.

For endothelial to monocyte adhesion assay, CellTracker Green CMFDA Dye (Invitrogen, catalog C2925) labeled THP-1 monocytes (ATCC) were added on HPAECs stimulated by hypoxia reoxygenation and IL-1β. After 90 minutes, floating cells were removed, and fluorescent imaging was performed with quantification of the adhered monocytes using Fiji ImageJ.

#### Lactate measurements.

HPAECs were treated with vehicle (DMSO) or syrosingopine for 24 hours. Then cell media were collected, and lactate levels were measured by a Lactate Assay Kit (Sigma, catalog MAK064) following the kit’s instructions.

### Statistics

The data were expressed as mean values ± standard deviation of mean (SEM). Two-group comparison was performed by unpaired 2-tailed Student’s *t* test with Welch’s correction. Multigroup comparison was performed by 1-way ANOVA with Šidák’s multiple-comparison test using GraphPad Prism 9 (GraphPad Software). *P* < 0.05 was considered statistically significant. scRNA-Seq and snRNA-Seq analyses were performed using the programs R (http://cran.r-project.org/), RStudio (https://posit.co/download/rstudio-desktop/) (https://posit.co/products/open-source/rstudio/), and Seurat (version 4.3.0).

### Study approval

All animal studies were conducted in accordance with NIH guidelines for the use and care of live animals and were approved by the Institutional Animal Care and Use Committees at University of Kansas and at Northwestern University.

### Data availability

The scRNA-Seq data were deposited in the NCBI’s Gene Expression Omnibus database (GEO GSE244064). Values for all data points in graphs are reported in the Supporting Data values file.

## Author contributions

RT and PPK conceived the study and designed the experiments. RT, RS, GR, GSB, SYA, JOS, MS, GC, and PPK performed experiments. RT, RS, GR, GSB, SYA, JOS, MS, GC, VD, YZ, BRT, MJS, and PPK analyzed and interpreted data. SEQ, EBT, and NSC provided input on experimental design. PPK and RT wrote the manuscript and all authors approved and commented on the manuscript.

## Supplementary Material

Supplemental data

Supplemental data set 1

Supplemental data set 2

Supplemental data set 3

Supplemental data set 4

Supplemental data set 5

Unedited blot and gel images

Supporting data values

## Version 1 12/02/2024

In-Press Preview

## Version 2 02/03/2025

Electronic publication
