# Supplementary material for: Postischemic inactivation of HIF prolyl hydroxylases in endothelium promotes maladaptive kidney repair by inducing glycolysis
Source: J Clin Invest. 2024 Dec 2;135(3):e176207. doi: 10.1172/JCI176207 (PMC11785929; doi:10.1172/JCI176207)
Supplement: Supplemental data [file jci-135-176207-s010.pdf]

Supplemental Materials for

Post-ischemic inactivation of HIF prolyl hydroxylases in endothelium promotes maladaptive kidney repair by inducing glycolysis

Tiwari et al.

The PDF file includes:
Supplemental Figures 1 to 15
Supplemental Tables 1 to 3
Supplemental References

Other Supplemental Material for this manuscript includes the following:

Supplemental Datasheets 1 to 5
- Supplemental Datasheet 1: DEGs of tubular cells
- Supplemental Datasheet 2: GSEA analysis of tubular cells
- Supplemental Datasheet 3: DEGs of mRECs and GSEA analysis
- Supplemental Datasheet 4: GSEA analysis of ECs from human samples (AKI vs control)
- Supplemental Datasheet 5: GSEA analysis of Mφ1

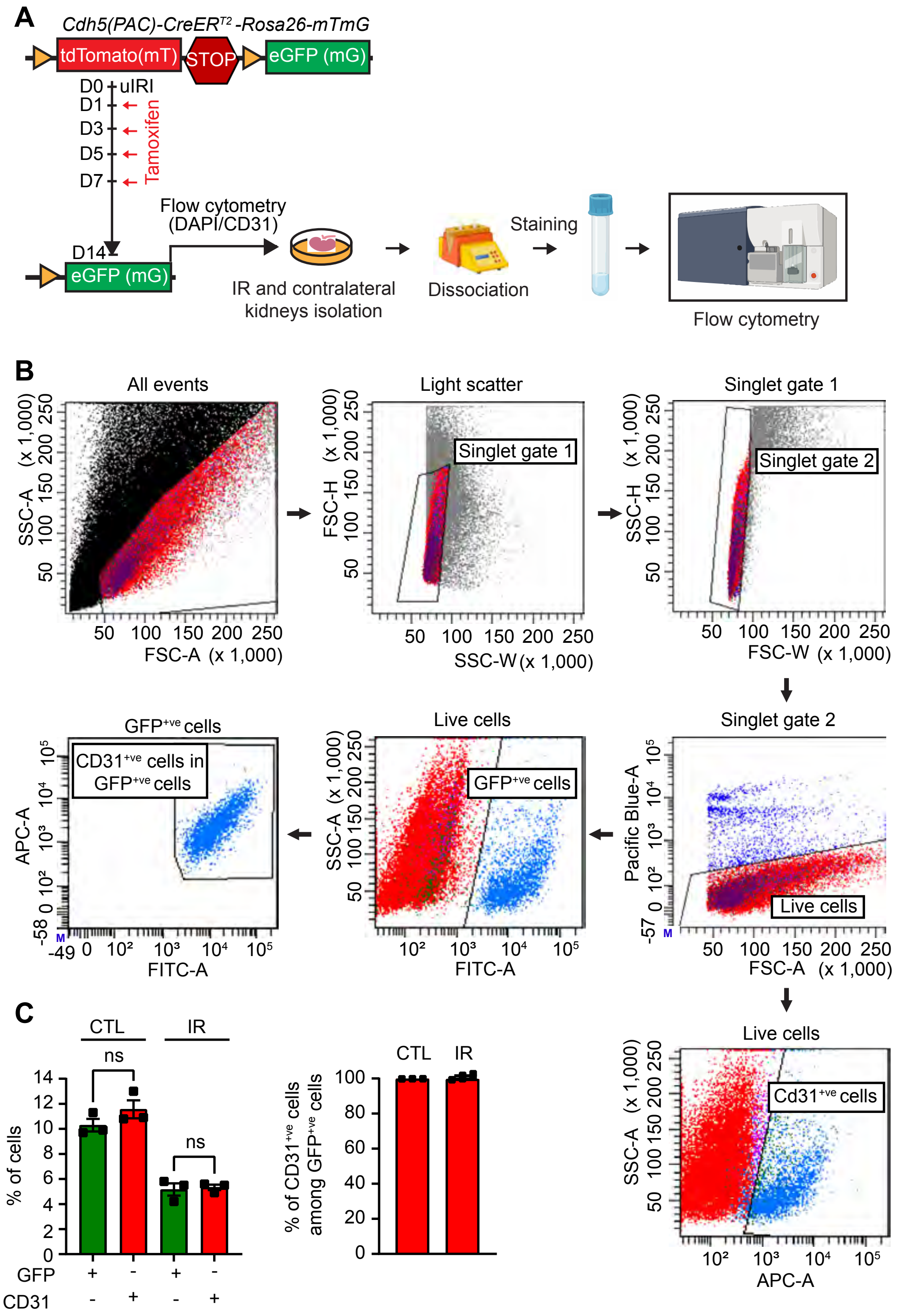

Supplemental Figure 1, associated with Figure 1. Efficiency of *Cre*- recombination in *Cdh5(PAC)-CreER^{T2}* transgenic mice following kidney IRI. (**A**) By crossing *Cdh5(PAC)-CreER^{T2}* mice with *ROSA26-ACTB-tdTomato,-EGFP (mTmG)* reporter mice, we generated *Cdh5(PAC)-CreER^{T2}; Rosa26-mTmG* mice, in which successful *Cre*- mediated excision is being indicated by GFP expression in endothelial cells (ECs). Mice were subjected to uIRI followed by tamoxifen administration starting at day 1 post uIRI to a total of 4 injections i.p. given every other day. The degree of EC-specific recombination was assessed by FACs analysis in single-cell suspensions prepared by CTL and IR kidneys. (**B**) Flow cytometric gating strategy used to define recombined ECs. Staining for the endothelial marker CD31 was used to detect ECs, while GFP identified cells with tamoxifen induced expression of *Cdh5-CreER^{T2}*. (**C**) Shown are the percentages of GFP^{+ve} and CD31^{+ve} cells (left panel) as well as the percentage of CD31^{+ve} cells among GFP^{+ve} cells (right panel) in CTL and IR kidneys at day 14 post uIRI (n=3). For (**C**), statistics were determined by one-way ANOVA with Sidak correction for multiple comparisons. ns, not statistically significant. ECs, endothelial cells; CTL, contralateral kidney; IR, kidney subjected to IRI.

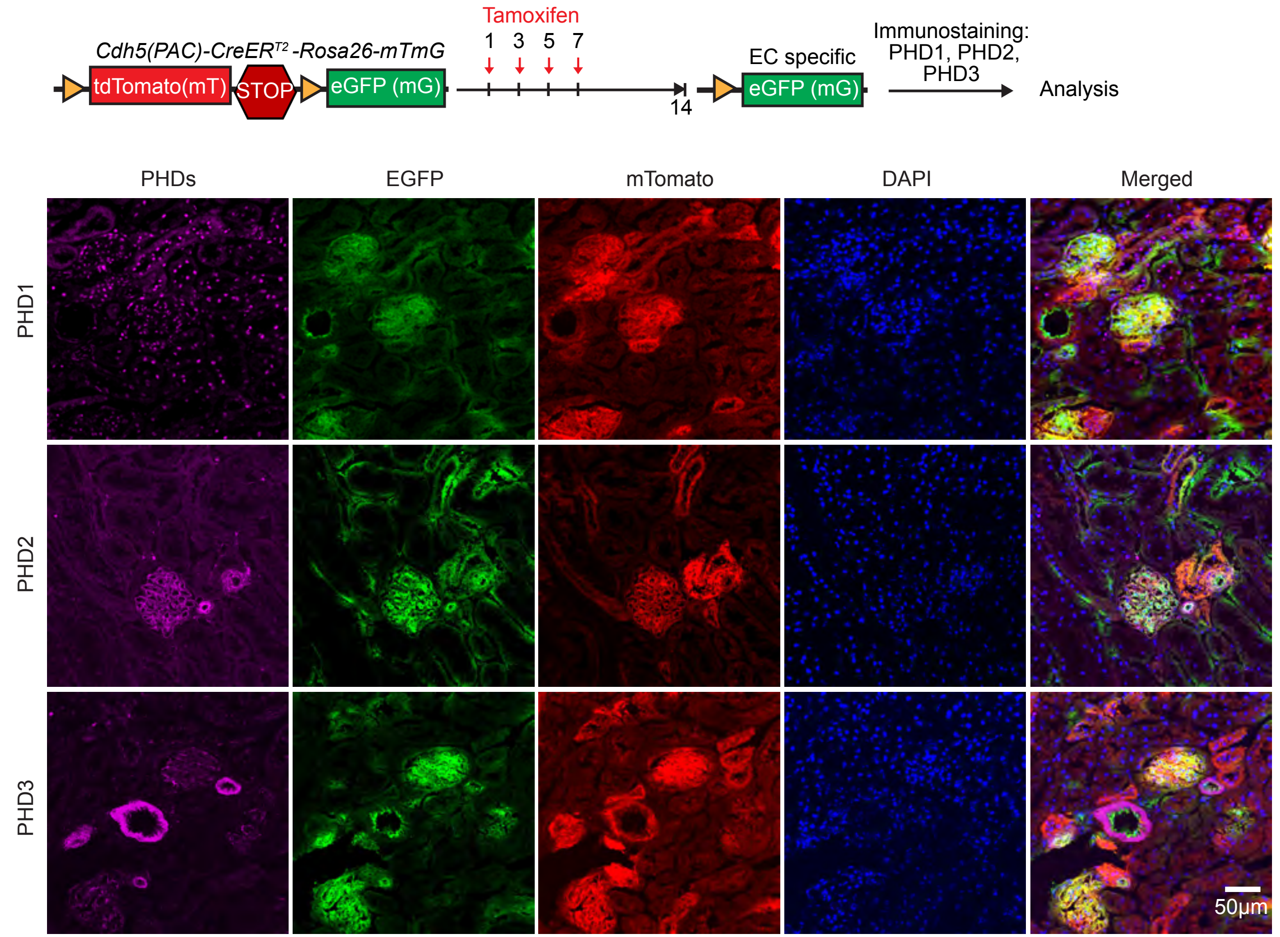

Supplemental Figure 2, associated with Figure 2. Murine kidney endothelium shows compartment-specific differences in the expression of PHD1, PHD2 and PHD3. Schematic view of experiment and representative images of immunofluorescence staining for PHD1, PHD2, PHD3 (magenta) and nuclear DAPI staining (blue) on kidney sections from *Cdh5(PAC)-CreER^{T2}-Rosa26mTmG* reporter mice after tamoxifen-induced recombination. Non recombined cells express membrane bound mTomato (red), whereas recombined cells express membrane-bound EGFP (green).

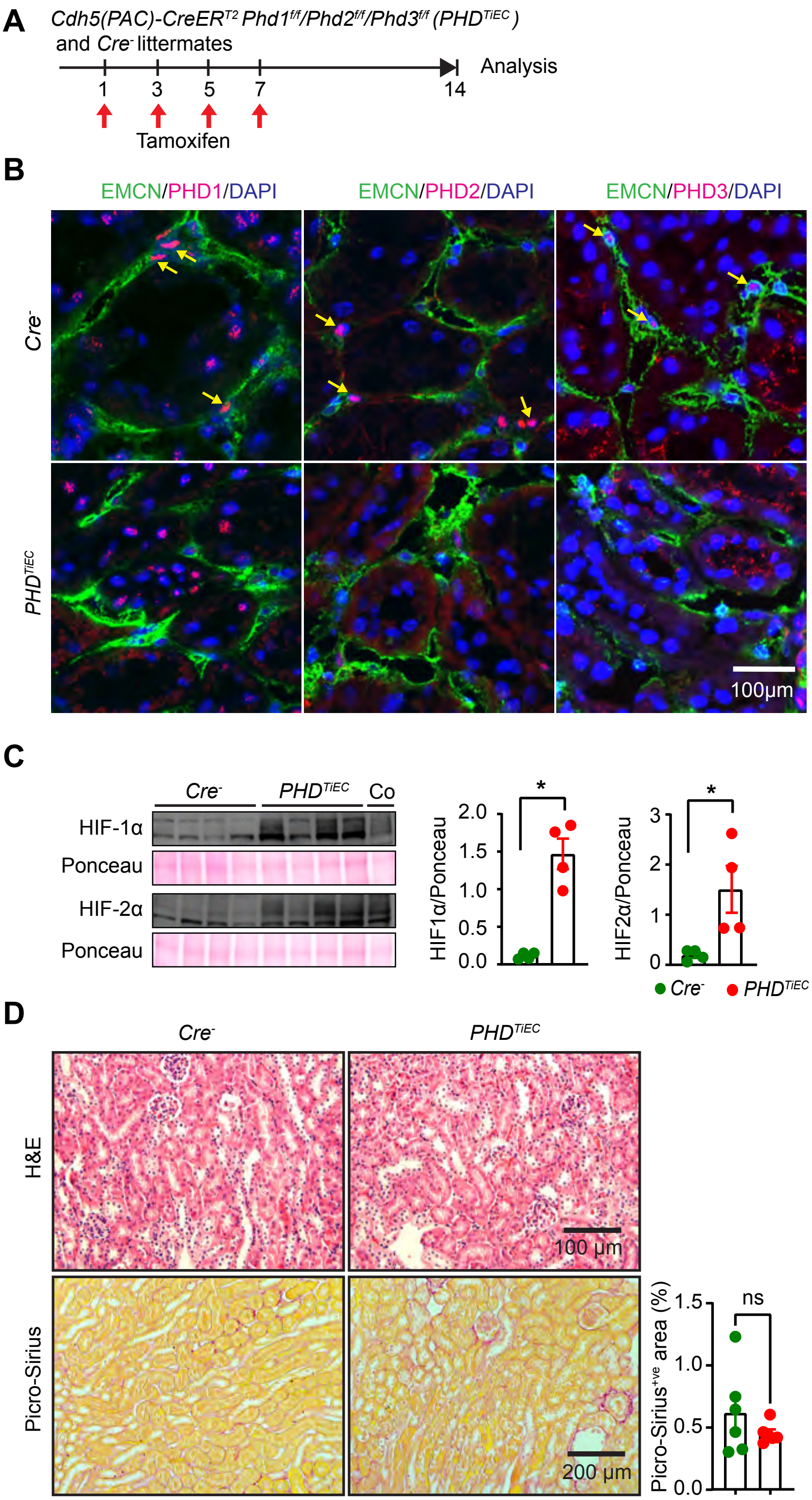

Supplemental Figure 3, associated with Figure 3. Simultaneous acute inactivation of endothelial PHD1, 2, and 3 stabilizes HIF without significantly affecting kidney morphology. (**A**) Schematic diagram depicting the tamoxifen administration regimen to *PHD*^{TiEC} and *Cre*$^-$ control mice followed by baseline analysis. (**B**) Immunofluorescence staining for PHD1-3 (red), EMCN (green) and nuclear DAPI (blue) using kidney sections from *PHD*^{TiEC} mice and *Cre*$^-$ controls collected 1 week after the last dose of tamoxifen. Arrows indicate positive staining. Scale bar, 100 µm. (**C**) Immunoblot analysis of HIF-1α and HIF-2α in kidney nuclear extracts isolated from *PHD*^{TiEC} mice and *Cre*$^-$ littermates. Co, positive control. (**D**) Representative images of H&E and Picro-Sirius red stained kidney sections for the indicated genotypes. Right side graph shows semi-quantitative analysis of Picro-Sirius red^{+ve} area for *PHD*^{TiEC} mice and *Cre*$^-$ littermates 1 week after the last dose of tamoxifen (n=4-6). Scale bars indicate 100 µm and 200 µm for H&E and Picro-Sirius red images, respectively. Data are represented as mean ± SEM. Statistics were determined by unpaired t- test with Welch's correction. *, $P < 0.05$; ns, not statistically significant. EMCN, endomucin.

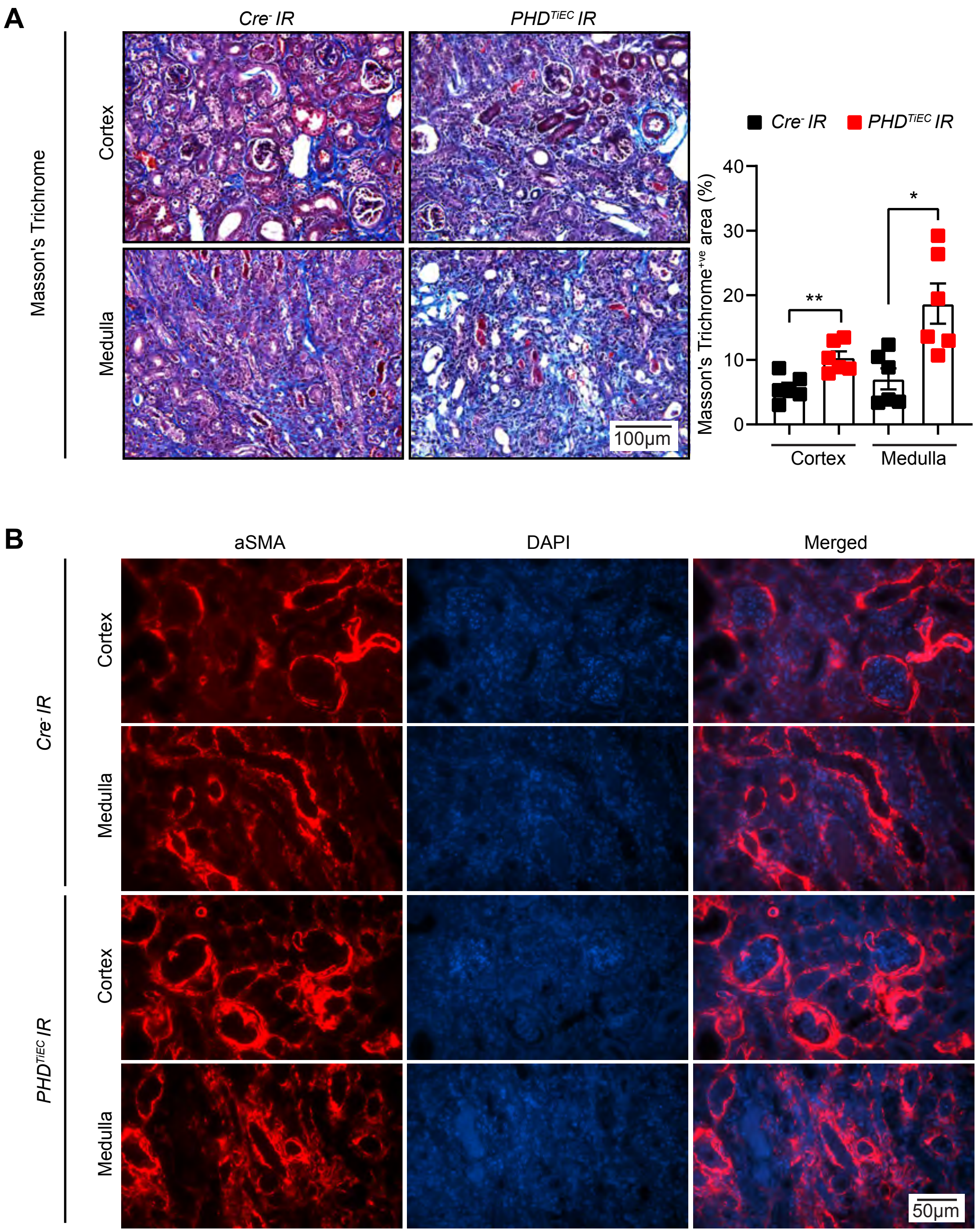

Supplemental Figure 4, associated with Figure 3. Post-ischemic simultaneous inactivation of endothelial PHD1, 2, and 3 promotes maladaptive kidney repair in unilateral ischemia reperfusion injury. *PHD*^{TiEC} mice and their *Cre*$^{-}$ littermates were subjected to 25 minutes of unilateral renal artery clamping. Treatment with tamoxifen was started on day 1 post uIRI involving 4 i.p. doses given every other day. Mice were sacrificed for molecular analysis on day 14 post uIRI. **(A)** Representative images of kidney cortex and medulla stained with Masson's trichrome as well semi-quantitative analysis of Masson's trichrome^{+ve} area on day 14 post-uIRI kidneys from *PHD*^{TiEC} mice and *Cre*$^{-}$ littermates. Scale bars indicate 100 µm. **(B)** Representative images of kidney cortex and medulla immunostained for α-smooth muscle actin (aSMA). Images were captured using a Nikon Ti2 Widefield fluorescence microscope. Scale bars indicate 50 µm. Statistics were determined by unpaired t- test with Welch's correction for cortex and medulla separately. n=6; *, $P < 0.05$; **, $P < 0.01$.

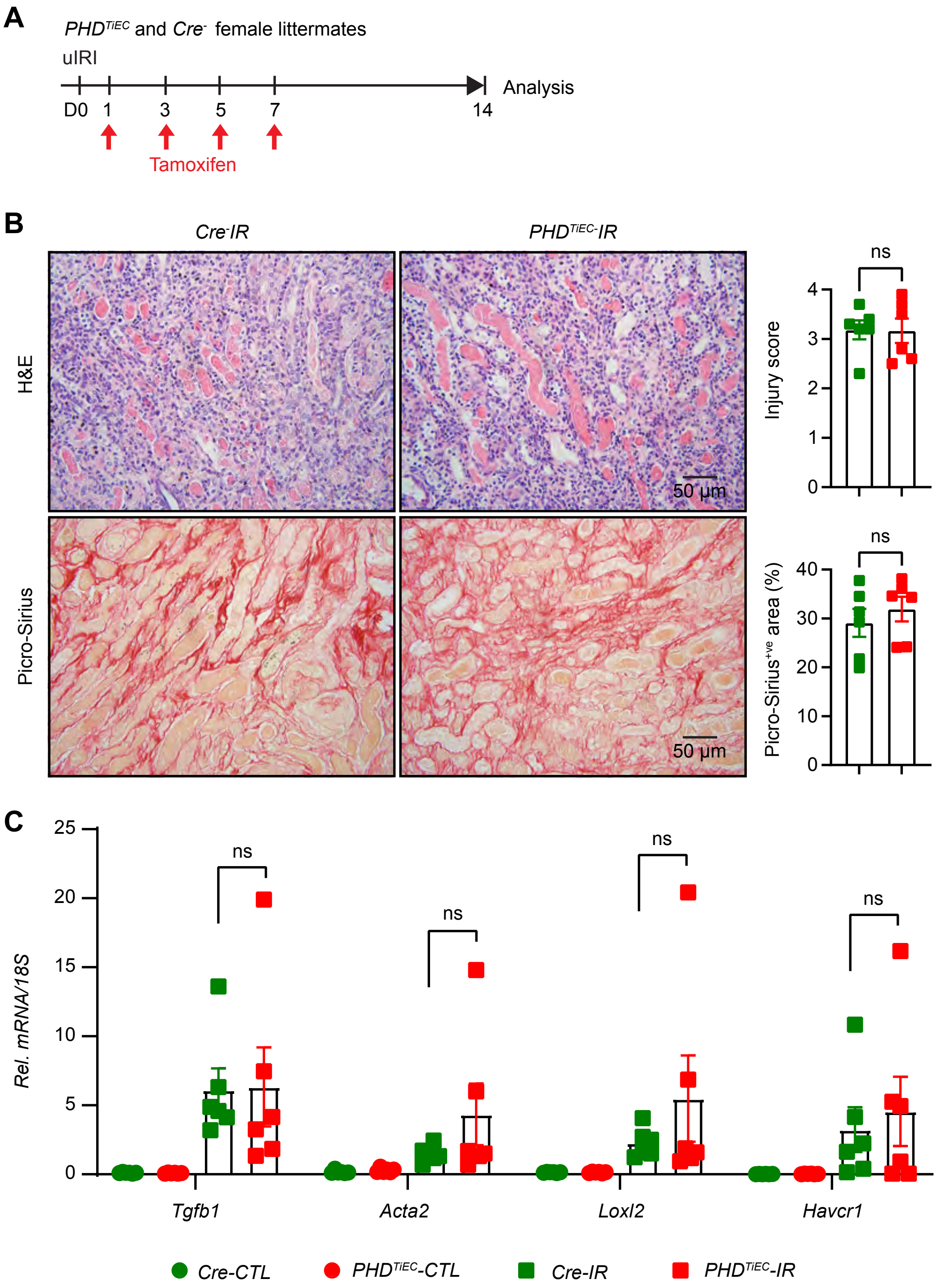

Supplemental Figure 5, associated with Figure 3. Post-ischemic inactivation of endothelial PHD1, 2, and 3 in female mice does not alter kidney repair. (A) Experimental scheme illustrates the experimental design. *PHD*^{TiEC} female mice and their *Cre*$^{-}$ female littermates were subjected to 30 minutes of unilateral renal artery clamping (uIRI). Treatment with tamoxifen was started on day 1 post uIRI involving 4 i.p. doses given every other day. Female mice were sacrificed for molecular analysis on day 14 post uIRI. **(B)** Representative images of H&E and Picro-Sirius red stained sections from day 14 post-ischemic kidneys of *PHD*^{TiEC} female mutants and their *Cre*$^{-}$ female littermates. Right panels show tubular injury score (Top) and semi-quantitative analysis of Picro-Sirius red^{+ve} area in the indicated genotypes. Scale bars indicate 50 µm. **(C)** mRNA levels of *Tgfb1*, *Acta2*, *Loxl2* and *Havcr1* in IR and CTL kidneys from *PHD*^{TiEC} female mice and their *Cre*$^{-}$ female controls at day 14 after uIRI. All bars show mean ± SEM (n=6). For (B), unpaired t- test with Welch's correction was used. For (C), statistics were determined using one-way ANOVA with Sidak correction for multiple comparisons. ns, not statistically significant. uIRI, unilateral IRI; CTL, contralateral; IR, kidney subjected to uIRI; Rel., relative.

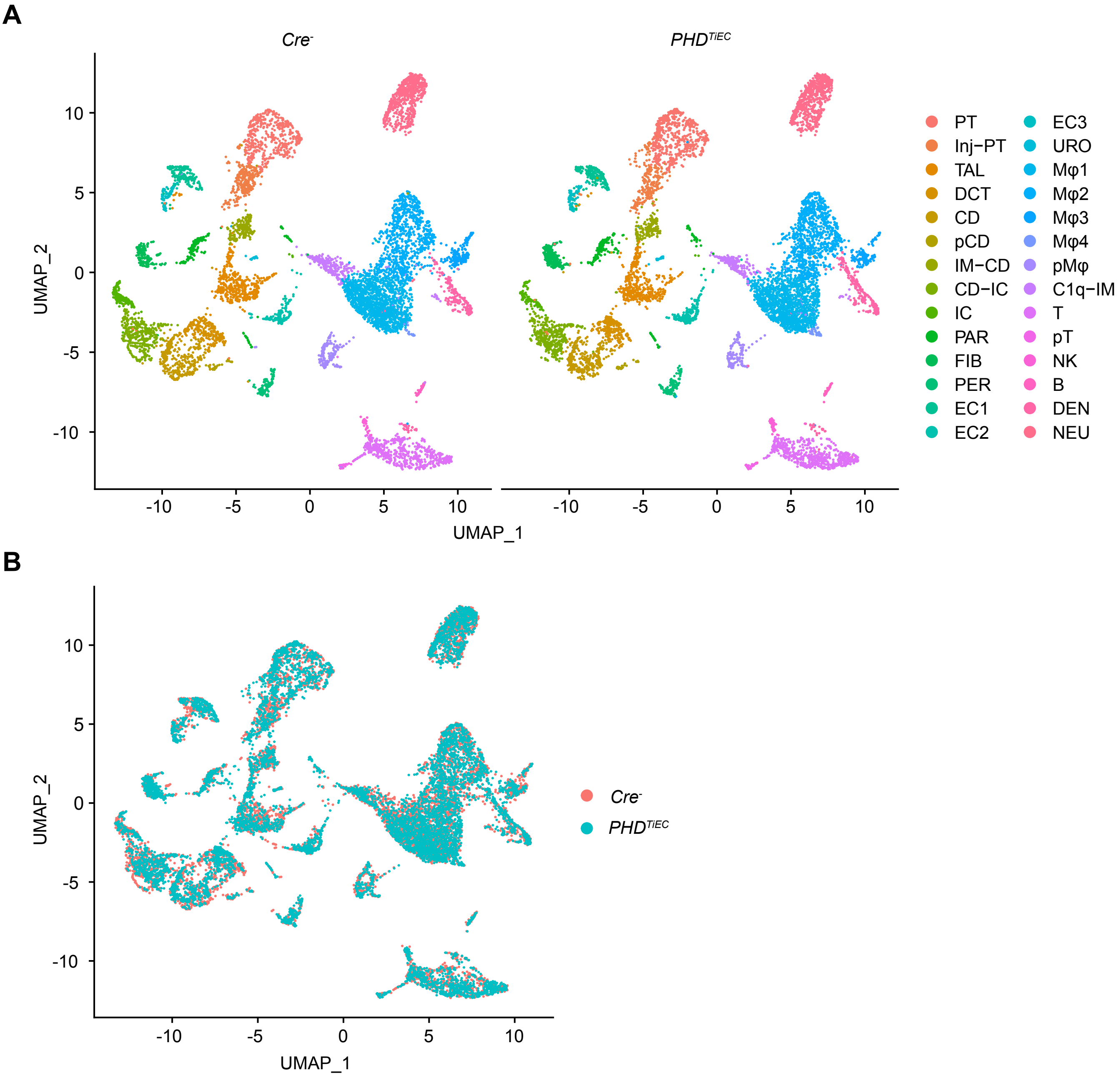

Supplemental Figure 6, associated with Figure 4. scRNA-seq analysis showing similar cell populations in day 14 post-ischemic kidneys of *PHD*^{TiEC} and *Cre*$^{-}$ mice. (**A**) UMAP showing different cell clusters in day 14 post-ischemic kidneys from *PHD*^{TiEC} and *Cre*$^{-}$ mice. (**B**) UMAP after overlaying samples of *Cre*$^{-}$ and *PHD*^{TiEC} day 14 post-ischemic kidneys showing similar clustering.

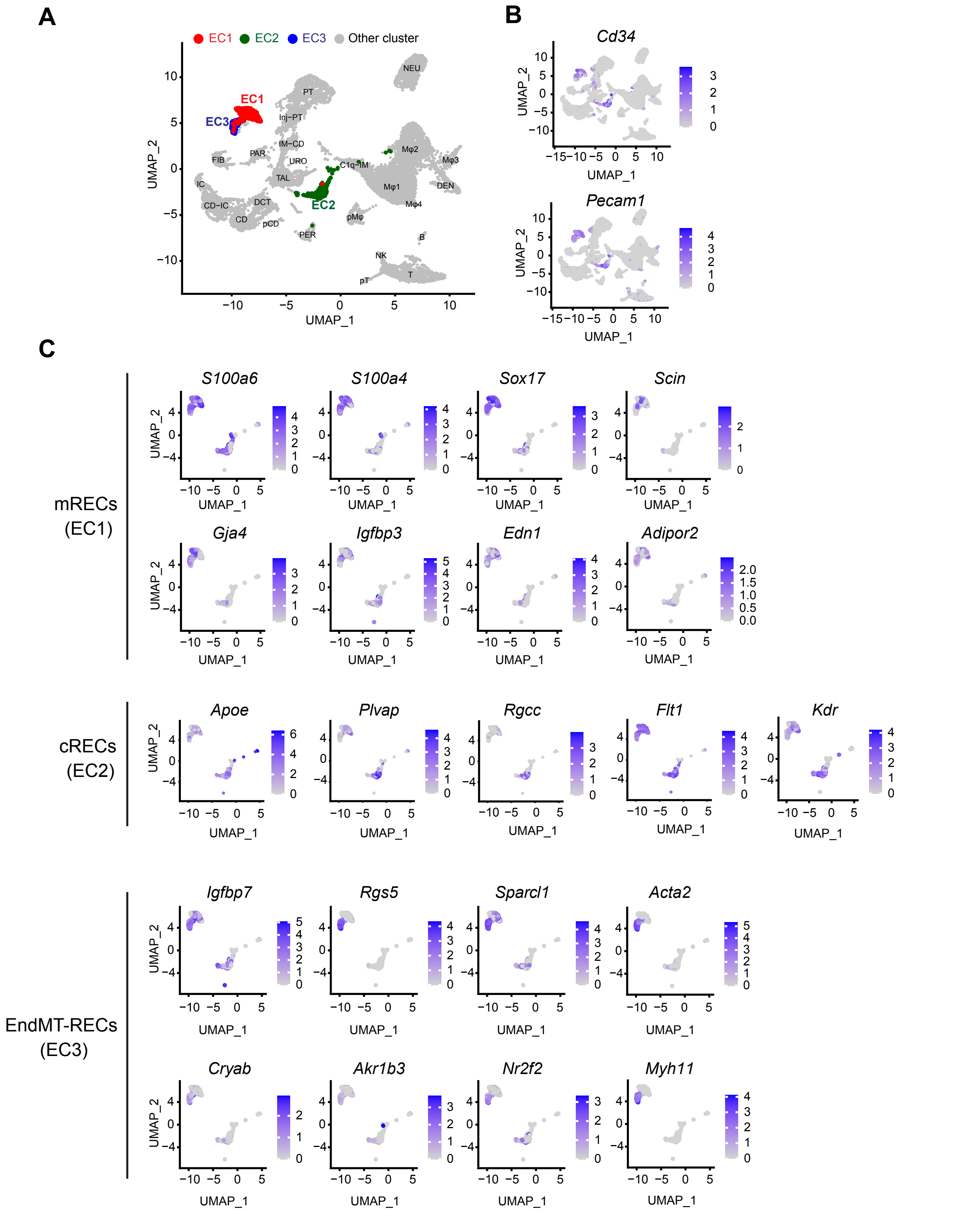

Supplemental Figure 7, associated with Figure 5. Marker genes used to identify different EC clusters. (**A**) UMAP plot with highlighted EC1 (red), EC2 (green) and EC3 (blue) cluster. (**B**) Feature plot showing expression of endothelial marker genes *Cd34* and *Pecam1* in EC1, EC2 and EC3 clusters. (**C**) Feature plot of EC marker genes used to identify mRECs (EC1), cRECs (EC2) and EndMT-RECs (EC3) clusters.

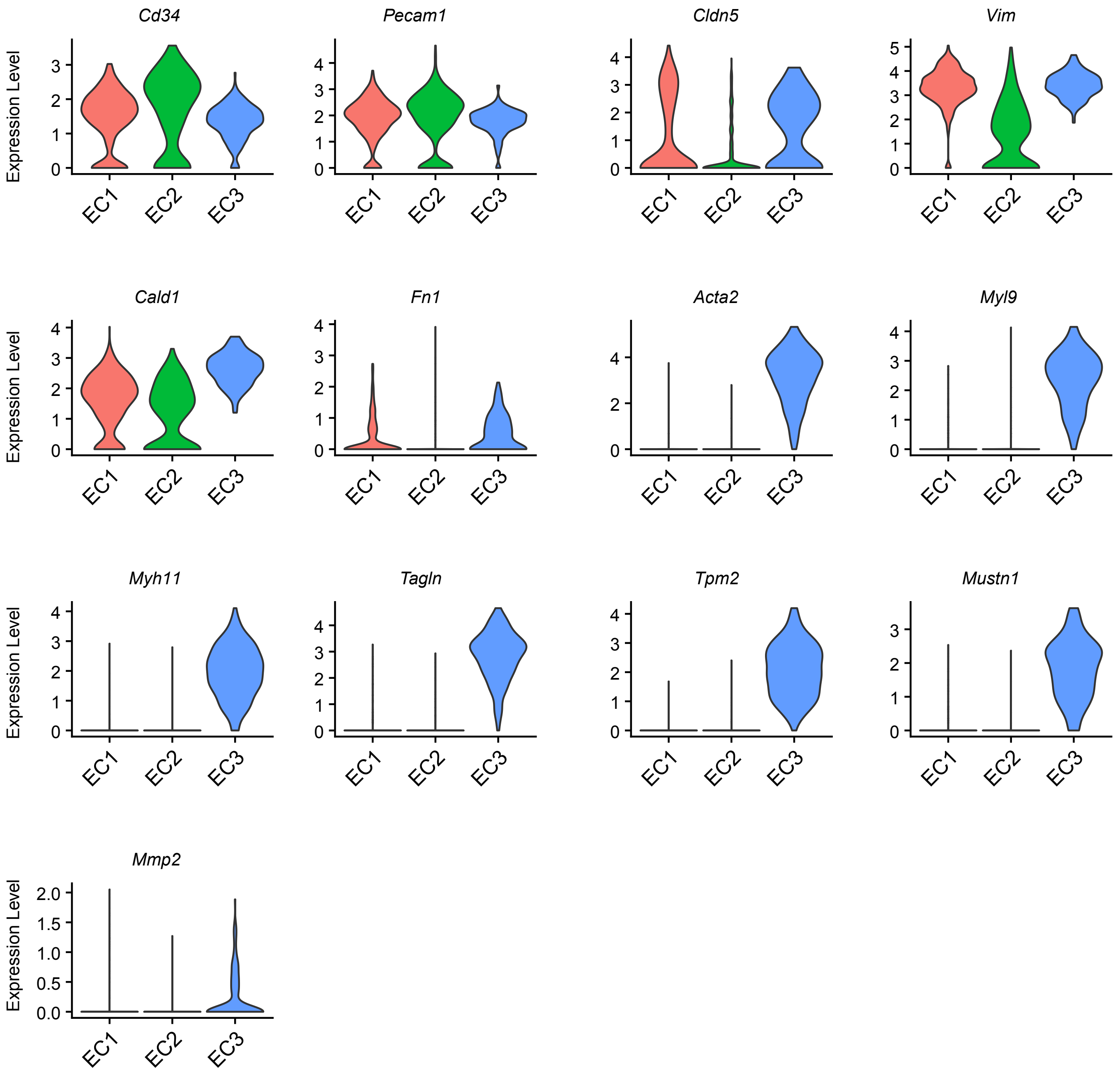

Supplemental Figure 8, associated with Figure 5. Violin plots showing the expression of endothelial and mesenchymal markers in EC1, EC2 and EC3 clusters. Violin plots show the expression of marker genes for ECs (*Cd34*, *Pecam1*, *Cldn5*) and mesenchymal cells (*Vim*, *Cald1*, *Fn1*, *Acta2*, *Myl9*, *Myh11*, *Tagln*, *Tpm2*, *Mustn1*, and *Mmp2*) in three EC clusters.

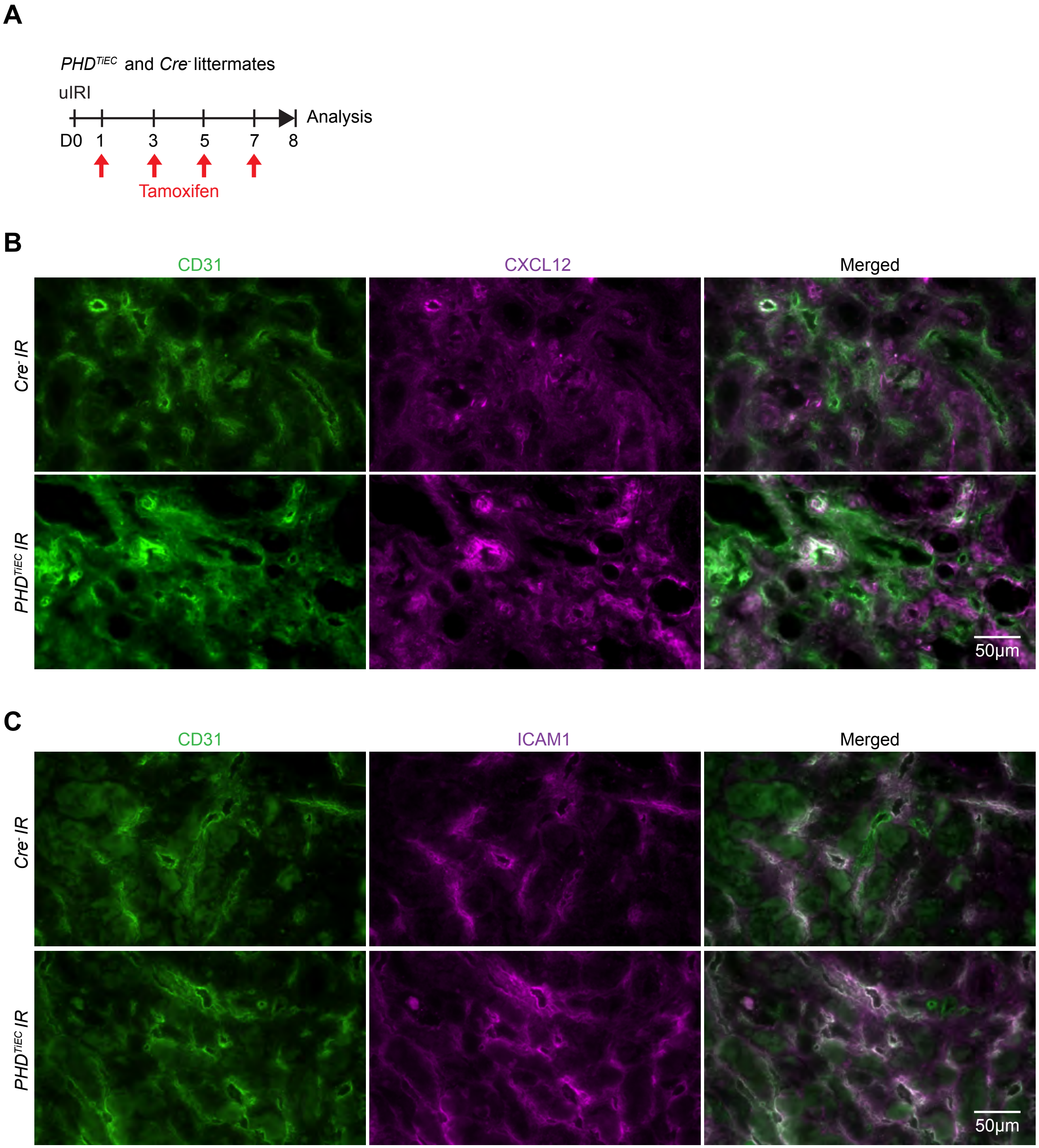

Supplemental Figure 9, associated with Figure 6. Post-ischemic simultaneous inactivation of endothelial PHD1, 2, and 3 induces the expression of CXCL12 and ICAM1 in kidney endothelial cells. (A) Scheme illustrating the experimental strategy applied. *PHD*^{TiEC} mice and their *Cre*$^{-}$ littermates were subjected to 25 minutes of unilateral renal artery clamping. Treatment with tamoxifen was started on day 1 post uIRI involving 4 i.p. doses given every other day. Mice were sacrificed for analysis on day 8 post uIRI. **(B)** and **(C)** Representative images of immunofluorescence staining for CXCL12 and ICAM1 (magenta) along with the endothelial marker CD31 (green) and nuclear DAPI staining (blue) of day 8 post-ischemic kidneys from *PHD*^{TiEC} and *Cre*$^{-}$ control mice. Images were captured using a Nikon Ti2 Widefield fluorescence microscope. Scale bar indicates 50 μm. IR, kidney subjected to IRI.

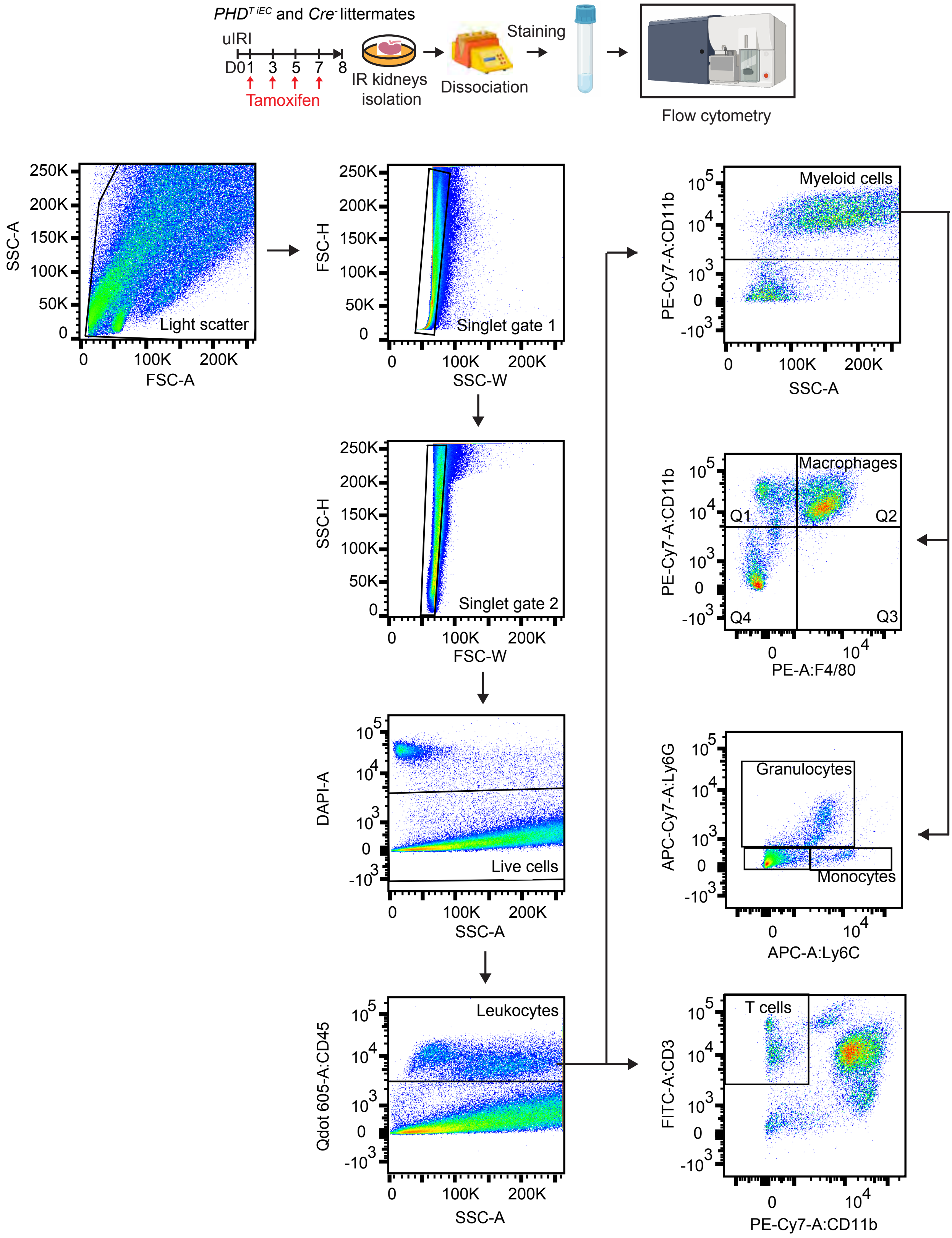

Supplemental Figure 10, associated with Figure 6. Strategy for the analysis of immune cells by flow cytometry. Schematic view of FACs experiment and gating strategy for the analysis of different immune cell populations in day 8 post-IRI kidneys from *PHD*^{TiEC} and *Cre*$^-$ mice. CD45$^+$ (leukocytes), CD45$^+$ CD3$^+$ (T cells), CD45$^+$ CD11b$^+$ (myeloid cells), CD45$^+$ CD11b$^+$ F4/80$^+$ (macrophages), CD45$^+$ CD11b$^+$ Ly6C$^+$ (monocytes), CD45$^+$ CD11b$^+$ Ly6G$^+$ (granulocytes) cells were measured.

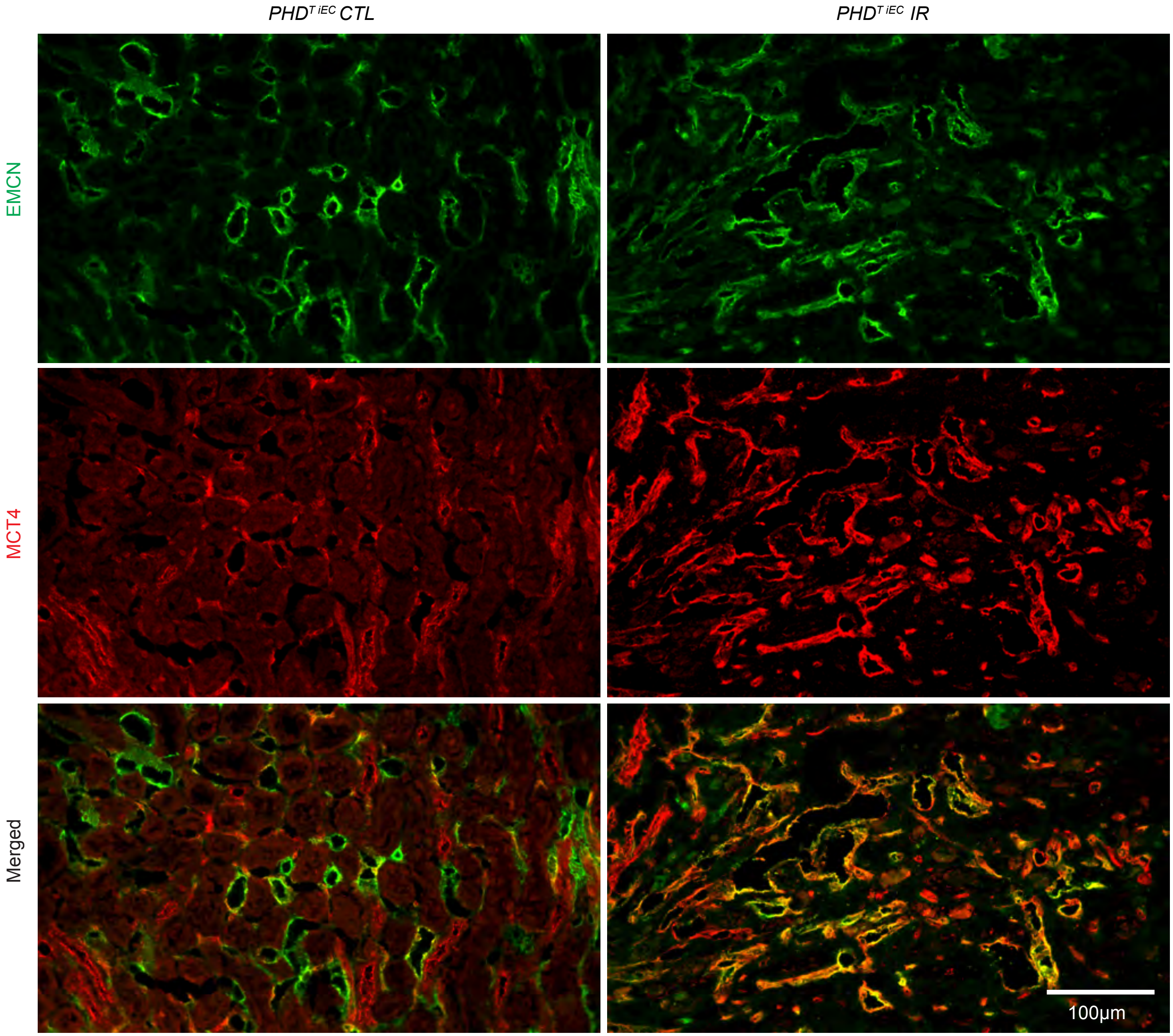

Supplemental Figure 11, associated with Figure 8. MCT4 expression in contralateral and day 14 post-ischemic kidneys of *PHD^{TiEC}* mouse . Representative images of immunofluorescence staining for MCT4 (red) and EMCN (green) of CTL and day 14 post-ischemic kidneys from *PHD^{TiEC}* mouse indicating increased expression of endothelial MCT4 following ischemic injury. Images were captured using Nikon a Ti2 Widefield fluorescence microscope. To ensure clarity, we disclose that the merged images shown here are also presented in Figure 8A. Scale bar,100 μm. CTL, contralateral kidney; IR, kidney subjected to uIRI.

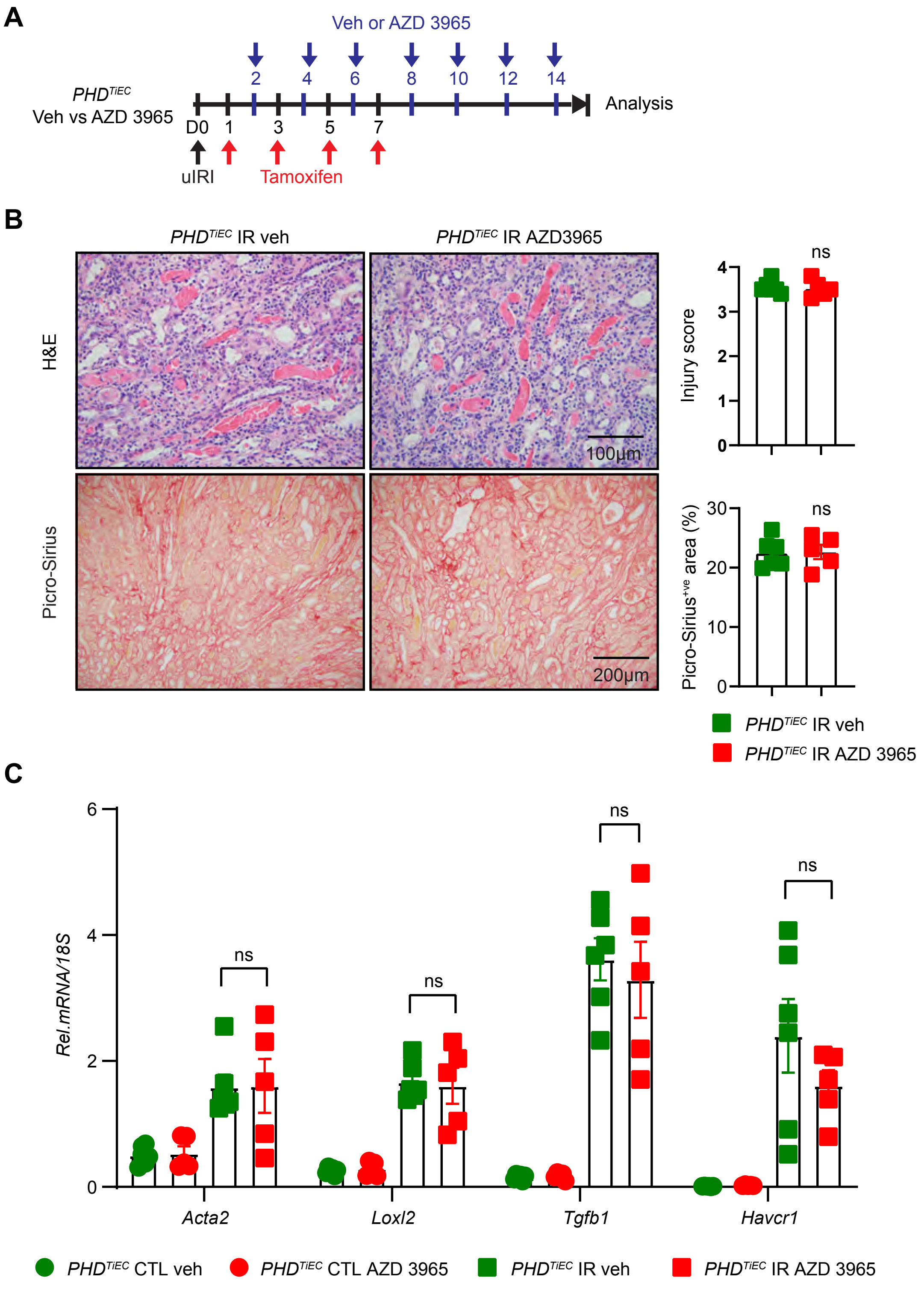

Supplemental Figure 12, associated with Figure 8. Post-ischemic treatment with MCT1 inhibitor AZD3965 does not alter post-ischemic kidney repair in *PHD^{TiEC}* mice. (A) Scheme shows the experimental protocol used. *PHD^{TiEC}* mice were subjected to 25 minutes of unilateral renal artery clamping. Tamoxifen was started on day 1 post uIRI and was given every other day until day 7 post uIRI. Treatment with AZD 3965 (30 mg/kg body weight, orally) was started at day 2 post uIRI and was given every other day until day 14, when mice were sacrificed for histopathological and molecular analysis. **(B)** Representative images of H&E and Picro-Sirius red stained day 14 post-ischemic kidneys from vehicle- vs AZD 3965-treated *PHD^{TiEC}* mutants. Right: Tubular injury score and semiquantitative analysis of Picro-Sirius red^{+ve} area of day 14 post-ischemic kidneys for the indicated experimental groups. All mice are *PHD^{TiEC}*. Scale bars indicate 100 µm and 200 µm for H&E and Picro-Sirius red images, respectively. **(C)** mRNA levels of *Acta2*, *Loxl2*, *Tgfb1* and *Havcr1* in CTL and IR kidneys from vehicle or AZD 3965-treated *PHD^{TiEC}* mice on day 14 after uIRI. Data are represented as mean ± SEM. For (**B**), statistics were determined by unpaired t- test with Welch's correction. For (**C**), statistics were determined using one-way ANOVA with Sidak correction for multiple comparisons. n=5-6. ns, not significant. uIRI, unilateral IRI; CTL, contralateral kidney; IR, kidney subjected to IRI; veh, vehicle.

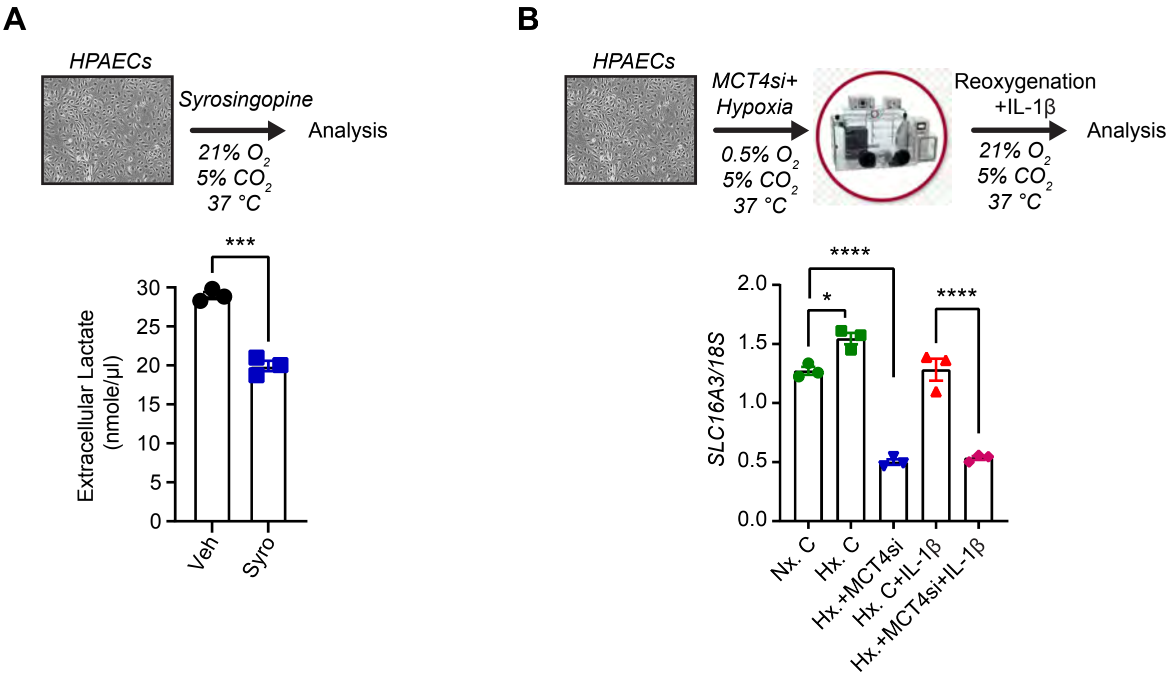

Supplemental Figure 13, associated with Figure 9. Effect of syrosingopine on extracellular lactate levels and knockdown efficiency of MCT4 siRNA. (**A**) HPAECs were treated for 24 hours with syrosingopine or vehicle (DMSO) and extracellular lactate levels were measured. (**B**) Experimental scheme for HPAECs subjected to 0.5% O_2 for 18 hours in the presence of MCT4 siRNA followed by reoxygenation for 8 hours in the presence of IL-1β (1 ng/ml). Graph showing *SLC16A3* (*MCT4*) mRNA levels in cells transfected with MCT4 siRNA compared to negative control siRNA transfected cells. All bars show mean ± SEM. For (**A**), unpaired t- test with Welch's correction was used. For (**B**), statistics were determined using one-way ANOVA with Sidak correction for multiple comparisons. *, P <0.05; ***, P <0.001; ****, P < 0.0001. Veh, vehicle; Syro, syrosingopine; Nx, Normoxia; Hx, Hypoxia/Reoxygenation; C, negative control siRNA; MCT4si, MCT4 siRNA.

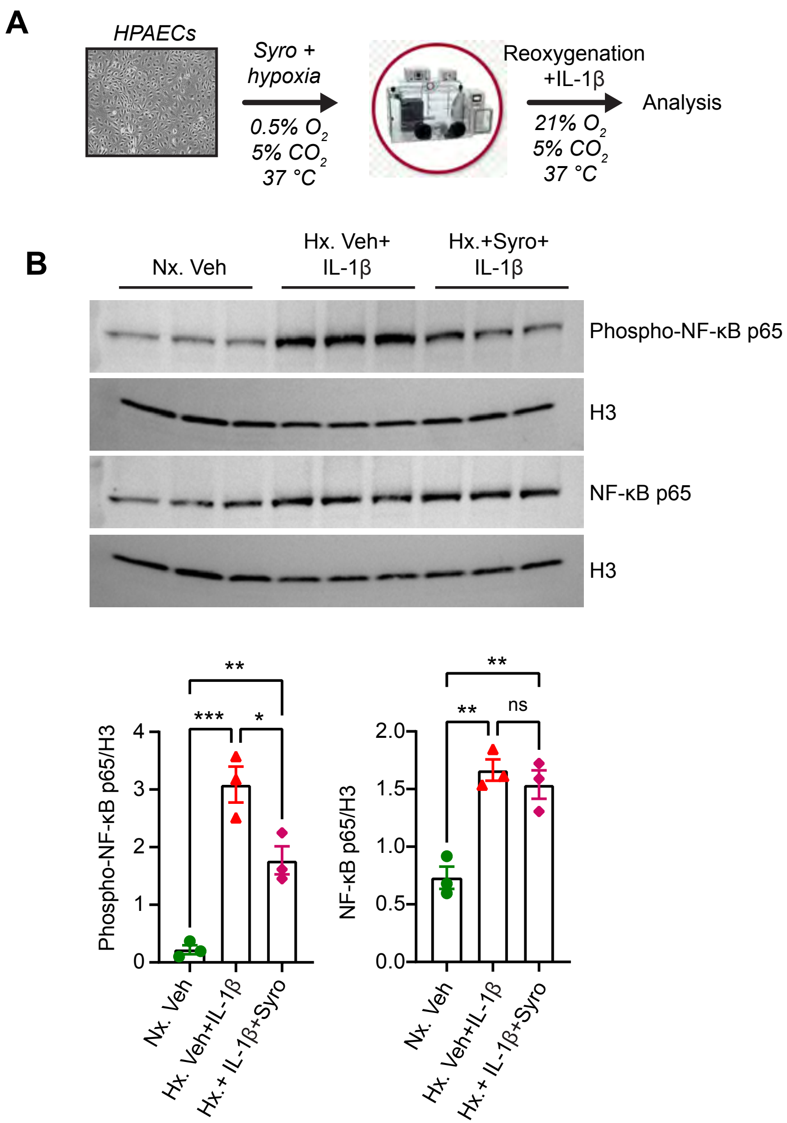

Supplemental Figure 14, associated with Figure 9. Syrosingopine reduced phospho-NF-κB p65 protein levels in HPAECs stimulated by hypoxia-reoxygenation and IL-1β. (A) Experimental scheme for HPAECs subjected to 0.5% O_2 for 18 hours in the presence of syrosingopine followed by reoxygenation for 8 hours in the presence of IL-1β (1 ng/ml) alog with a control group. (**B**) Immunoblot analysis of phospho-NF-κB p65 and total NF-κB p65 nuclear extracts isolated from HPAECs subjected to indicated conditions. Histone H3 levels were used as loading control. Data are represented as mean ± SEM. Statistics were determined using one-way ANOVA with Sidak correction for multiple comparisons. *, *P* <0.05; **, *P* <0.01; ***, *P* <0.001; ns, not significant; Nx, Normoxia; Hx, Hypoxia/Reoxygenation; Veh, vehicle; Syro, syrosingopine.

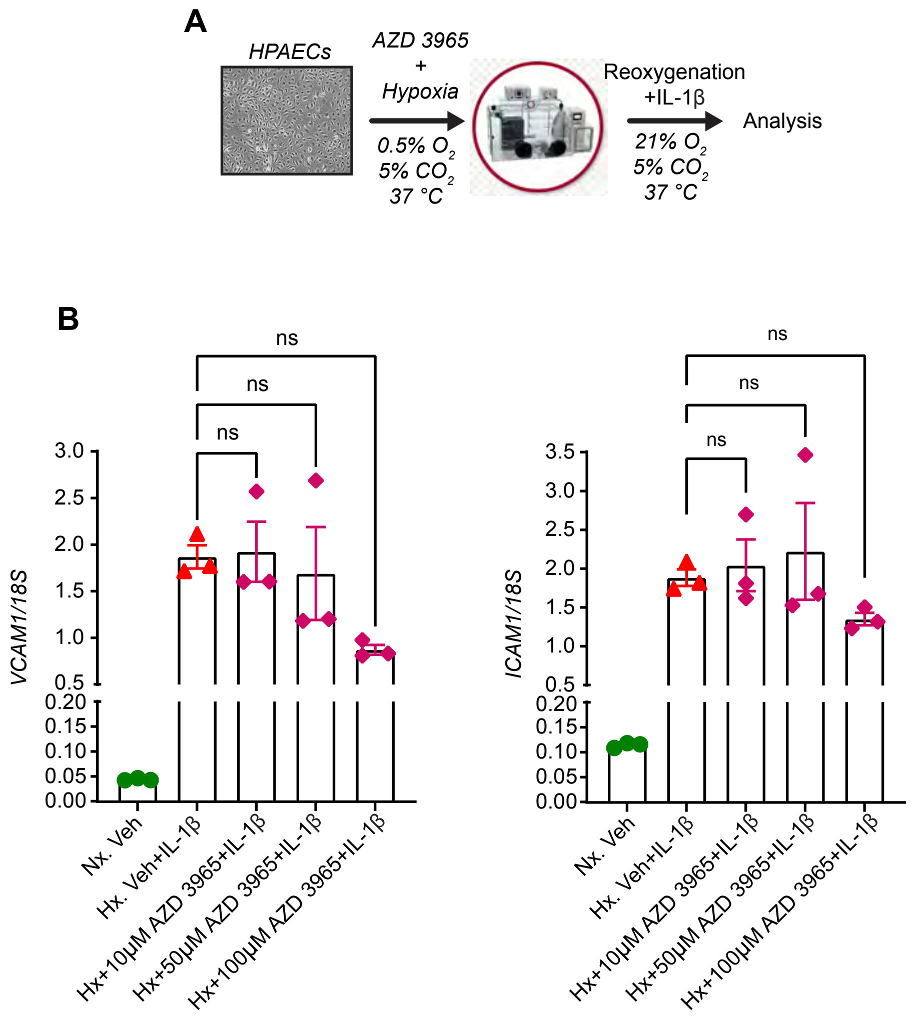

Supplemental Figure 15, associated with Figure 9. Treatment with the MCT1 inhibitor AZD3965 does not suppress inflammation in HPAECs activated by Hypoxia/Reoxygenation and IL-1β. (A) Experimental scheme for HPAECs subjected to 0.5% O_2 for 18 hours in the presence of AZD3965 (10, 50 and 100 μM) followed by reoxygenation for 8 hours in the presence of IL-1β (1 ng/ml). **(B)** mRNA levels of *VCAM1* and *ICAM1* in HPAECs, that were activated by Hypoxia/Reoxygenation and IL-1 β in the presence of different concentration of AZD3965. Data are represented as mean ± SEM. Statistics were determined by one-way ANOVA with Sidak correction for multiple comparisons. n=3; ns, not significant.

Supplemental Table 1. List of GEO numbers and details of samples which were used to assess the level of *Eglns* in human kidney ECs.

Samples	GEO	Source name	Organism	Subject status	Tissue
1	GSM4191941	Tumor-nephrectory	Homo sapiens	Normal/healthy	Adult kidney
2	GSM4191942	Tumor-nephrectory	Homo sapiens	Normal/healthy	Adult kidney
3	GSM4191943	Tumor-nephrectory	Homo sapiens	Normal/healthy	Adult kidney
4	GSM4191944	Tumor-nephrectory	Homo sapiens	Normal/healthy	Adult kidney
5	GSM4191945	Tumor-nephrectory	Homo sapiens	Normal/healthy	Adult kidney
6	GSM4191946	Tumor-nephrectory	Homo sapiens	Normal/healthy	Adult kidney
7	GSM4191947	Tumor-nephrectory	Homo sapiens	Normal/healthy	Adult kidney
8	GSM4191948	Tumor-nephrectory	Homo sapiens	Normal/healthy	Adult kidney
9	GSM4191949	Tumor-nephrectory	Homo sapiens	Normal/healthy	Adult kidney
10	GSM4191950	Tumor-nephrectory	Homo sapiens	Normal/healthy	Adult kidney
11	GSM4191951	Tumor-nephrectory	Homo sapiens	Normal/healthy	Adult kidney
12	GSM4191952	Living donor	Homo sapiens	Normal/healthy	Adult kidney
13	GSM4191953	Living donor	Homo sapiens	Normal/healthy	Adult kidney

14	GSM4191954	Living donor	Homo sapiens	Normal/healthy	Adult kidney
15	GSM4191955	Tumor-nephrectory	Homo sapiens	Normal/healthy	Adult kidney
16	GSM4191956	Tumor-nephrectory	Homo sapiens	Normal/healthy	Adult kidney
17	GSM4191957	Tumor-nephrectory	Homo sapiens	Normal/healthy	Adult kidney
18	GSM4191958	Tumor-nephrectory	Homo sapiens	Normal/healthy	Adult kidney
19	GSM4191959	Tumor-nephrectory	Homo sapiens	Normal/healthy	Adult kidney
20	GSM4191960	Surveillance biopsy	Homo sapiens	Normal/healthy	Adult kidney
21	GSM4191961	Surveillance biopsy	Homo sapiens	Normal/healthy	Adult kidney
22	GSM4191962	Surveillance biopsy	Homo sapiens	Normal/healthy	Adult kidney
23	GSM4191963	Surveillance biopsy	Homo sapiens	Normal/healthy	Adult kidney
24	GSM4191964	Surveillance biopsy	Homo sapiens	Normal/healthy	Adult kidney

Supplemental Table 2. List of used marker genes to identify different cell cluster of scRNA seq data of *Cre*$^{-}$ and *PHD*^{TiEC} mice and their references.

Marker genes	Cell cluster	Reference
Pck1, Lrp2, Slc5a12, Slc34a1	Proximal tubule (PT)	(1, 2)
Havcr1	Injured PT (Inj-PT)	(1)
Umod, Slc12a1	Thick ascending limb (TAL)	(1)
Slc12a3	Distal convoluted tubule (DCT)	(3)
Fxyd4, Aqp2	Collecting duct (CD)	(4)
Slc14a2	Inner medullary collecting duct (IM-CD)	(5)
Atp6v1g3, Scl26a4	Intercalated cells (IC)	(1, 6)
Ncam1	Parietal cells (PAR)	(7)
Col1a2, Dcn	Fibroblasts (FIB)	(2, 8, 9)
Myh11, Acta2,	Pericytes (PER)/smooth muscle cells	(9)
Cd34, Pecam1, Igfbp3	Endothelial cells (EC)	(1, 10)
Upk1b	Urothelial cells (URO)	(11)
Mki67, Top2a	Proliferating cells (p)	(1) (12)
Ptprc	Immune cells	(6)
Itgam	Myeloid cells	(12)
Adgre1, Cx3cr1	Macrophages (Mφ)	(1, 12)
C1qa, C1qb, C1qc	C1qa, C1qb and C1qc expressing immune cell (C1q-IM); potentially resident macrophage/monocyte	(13, 14)
Trbc2, Skap1	T cells	(14, 15)
GZMA, GZMB	Natural killer cells (NK cells)	(16)
Ms4a1, Pax5, Bcl11a	B cells	(15, 17-19)
Flt3	Dendritic cells (DC)	(20, 21)
S100a8, S100a9	Neutrophils (NEU)	(22)

Supplemental Table 3. Primer sequences. Shown are sequences of primer sets used for the expression analysis of the indicated mouse and human genes by RT-PCR.

Gene	Forward Primer	Reverse Primer
**Mouse**		
Loxl2	5′-GATCTTCAGCCCCGATGGA-3′	5′-CAAGGGTTGCTCTGGCTTGT-3′
Tgfb1	5′-TGGCGAGCCTTAGTTTGGA-3′	5′-TCGACATGGAGCTGGTGAAA-3′
Acta2	5′-CCTGACGCTGAAGTATCCGATAG-3′	5′-TTTTCCATGTCGTCCCAGTTG-3′
Havcr1	5′-AAACCAGAGATTCCCACACG-3′	5′-GTCGTGGGTCTTCCTGTAGC-3′
**Human**		
SLC16A3	5′-GGGTGGGAACCGTGTCATT-3′	5′-CTTGCGGCTTGGCTTCA-3′
VCAM1	5′-GCTTCAGGAGCTGAATACCC-3′	5′-AAGGATCACGACCATCTTCC-3′
ICAM1	5′-CCACAGTCACCTATGGCAAC-3′	5′-AGTGTCTCCTGGCTCTGGTT-3′

Supplemental References

1. Xu L, Guo J, Moledina DG, and Cantley LG. Immune-mediated tubule atrophy promotes acute kidney injury to chronic kidney disease transition. *Nat Commun.* 2022;13(1):4892.
2. Rudman-Melnick V, Adam M, Potter A, Chokshi SM, Ma Q, Drake KA, et al. Single-Cell Profiling of AKI in a Murine Model Reveals Novel Transcriptional Signatures, Profibrotic Phenotype, and Epithelial-to-Stromal Crosstalk. *J Am Soc Nephrol.* 2020;31(12):2793-814.
3. Miao Z, Balzer MS, Ma Z, Liu H, Wu J, Shrestha R, et al. Single cell regulatory landscape of the mouse kidney highlights cellular differentiation programs and disease targets. *Nat Commun.* 2021;12(1):2277.
4. Klocke J, Kim SJ, Skopnik CM, Hinze C, Boltengagen A, Metzke D, et al. Urinary single-cell sequencing captures kidney injury and repair processes in human acute kidney injury. *Kidney Int.* 2022;102(6):1359-70.
5. Ransick A, Lindstrom NO, Liu J, Zhu Q, Guo JJ, Alvarado GF, et al. Single-Cell Profiling Reveals Sex, Lineage, and Regional Diversity in the Mouse Kidney. *Dev Cell.* 2019;51(3):399-413 e7.
6. McEvoy CM, Murphy JM, Zhang L, Clotet-Freixas S, Mathews JA, An J, et al. Single-cell profiling of healthy human kidney reveals features of sex-based transcriptional programs and tissue-specific immunity. *Nat Commun.* 2022;13(1):7634.
7. Liu WB, Huang GR, Liu BL, Hu HK, Geng J, Rui HL, et al. Single cell landscape of parietal epithelial cells in healthy and diseased states. *Kidney Int.* 2023;104(1):108-23.
8. Muhl L, Genove G, Leptidis S, Liu J, He L, Mocci G, et al. Single-cell analysis uncovers fibroblast heterogeneity and criteria for fibroblast and mural cell identification and discrimination. *Nat Commun.* 2020;11(1):3953.

9. Kirita Y, Wu H, Uchimura K, Wilson PC, and Humphreys BD. Cell profiling of mouse acute kidney injury reveals conserved cellular responses to injury. *Proc Natl Acad Sci U S A.* 2020;117(27):15874-83.

10. Dumas SJ, Meta E, Borri M, Goveia J, Rohlenova K, Conchinha NV, et al. Single-Cell RNA Sequencing Reveals Renal Endothelium Heterogeneity and Metabolic Adaptation to Water Deprivation. *J Am Soc Nephrol.* 2020;31(1):118-38.

11. Tanigawa S, Tanaka E, Miike K, Ohmori T, Inoue D, Cai CL, et al. Generation of the organotypic kidney structure by integrating pluripotent stem cell-derived renal stroma. *Nat Commun.* 2022;13(1):611.

12. Conway BR, O'Sullivan ED, Cairns C, O'Sullivan J, Simpson DJ, Salzano A, et al. Kidney Single-Cell Atlas Reveals Myeloid Heterogeneity in Progression and Regression of Kidney Disease. *J Am Soc Nephrol.* 2020;31(12):2833-54.

13. Zimmerman KA, Bentley MR, Lever JM, Li Z, Crossman DK, Song CJ, et al. Single-Cell RNA Sequencing Identifies Candidate Renal Resident Macrophage Gene Expression Signatures across Species. *J Am Soc Nephrol.* 2019;30(5):767-81.

14. Wu H, Malone AF, Donnelly EL, Kirita Y, Uchimura K, Ramakrishnan SM, et al. Single-Cell Transcriptomics of a Human Kidney Allograft Biopsy Specimen Defines a Diverse Inflammatory Response. *J Am Soc Nephrol.* 2018;29(8):2069-80.

15. Li H, Qu L, Yang Y, Zhang H, Li X, and Zhang X. Single-cell Transcriptomic Architecture Unraveling the Complexity of Tumor Heterogeneity in Distal Cholangiocarcinoma. *Cell Mol Gastroenterol Hepatol.* 2022;13(5):1592-609 e9.

16. Bezman NA, Kim CC, Sun JC, Min-Oo G, Hendricks DW, Kamimura Y, et al. Molecular definition of the identity and activation of natural killer cells. *Nat Immunol.* 2012;13(10):1000-9.

17. Morgan D, and Tergaonkar V. Unraveling B cell trajectories at single cell resolution. *Trends Immunol.* 2022;43(3):210-29.

18. Liu Y, Hu J, Liu D, Zhou S, Liao J, Liao G, et al. Single-cell analysis reveals immune landscape in kidneys of patients with chronic transplant rejection. *Theranostics.* 2020;10(19):8851-62.

19. Qu J, Yang F, Zhu T, Wang Y, Fang W, Ding Y, et al. A reference single-cell regulomic and transcriptomic map of cynomolgus monkeys. *Nat Commun.* 2022;13(1):4069.

20. Villani AC, Satija R, Reynolds G, Sarkizova S, Shekhar K, Fletcher J, et al. Single-cell RNA-seq reveals new types of human blood dendritic cells, monocytes, and progenitors. *Science.* 2017;356(6335).

21. Arazi A, Rao DA, Berthier CC, Davidson A, Liu Y, Hoover PJ, et al. The immune cell landscape in kidneys of patients with lupus nephritis. *Nat Immunol.* 2019;20(7):902-14.

22. Xie X, Shi Q, Wu P, Zhang X, Kambara H, Su J, et al. Single-cell transcriptome profiling reveals neutrophil heterogeneity in homeostasis and infection. *Nat Immunol.* 2020;21(9):1119-33.
