# Supplementary material for: Postischemic inactivation of HIF prolyl hydroxylases in endothelium promotes maladaptive kidney repair by inducing glycolysis
Source: J Clin Invest. 2024 Dec 2;135(3):e176207. doi: 10.1172/JCI176207 (PMC11785929; doi:10.1172/JCI176207)
Supplement: Unedited blot and gel images [file jci-135-176207-s016.pdf]

# Full unedited gel for Supplemental Figure 3 C

**HIF-1*α***

**04_16_2018 Western blot P1P2P3 Vei-ve Vs Ve-+ve CTL Nuclear Extract (40µg).**

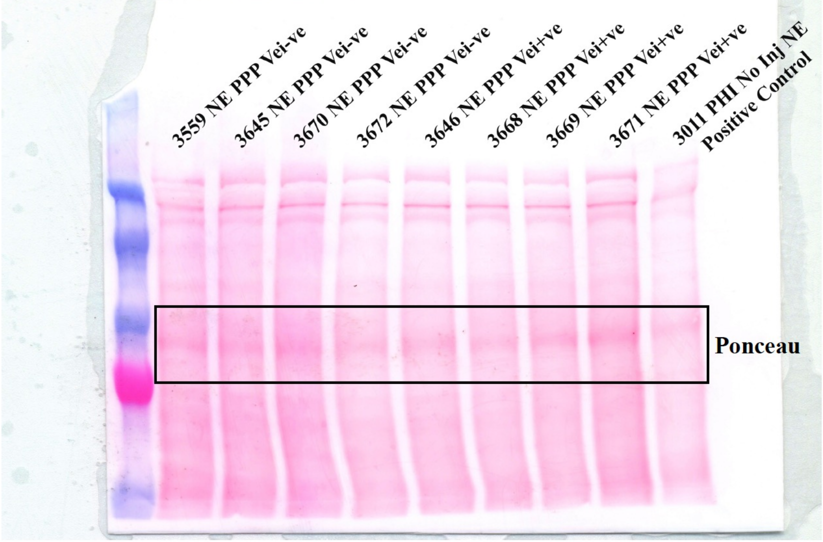


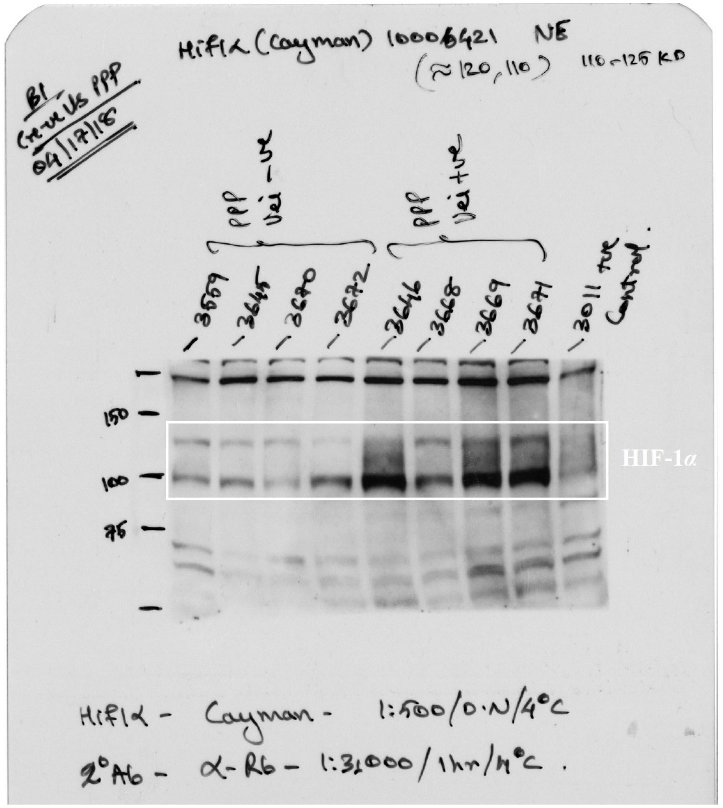

# Full unedited gel for Supplemental Figure 3 C

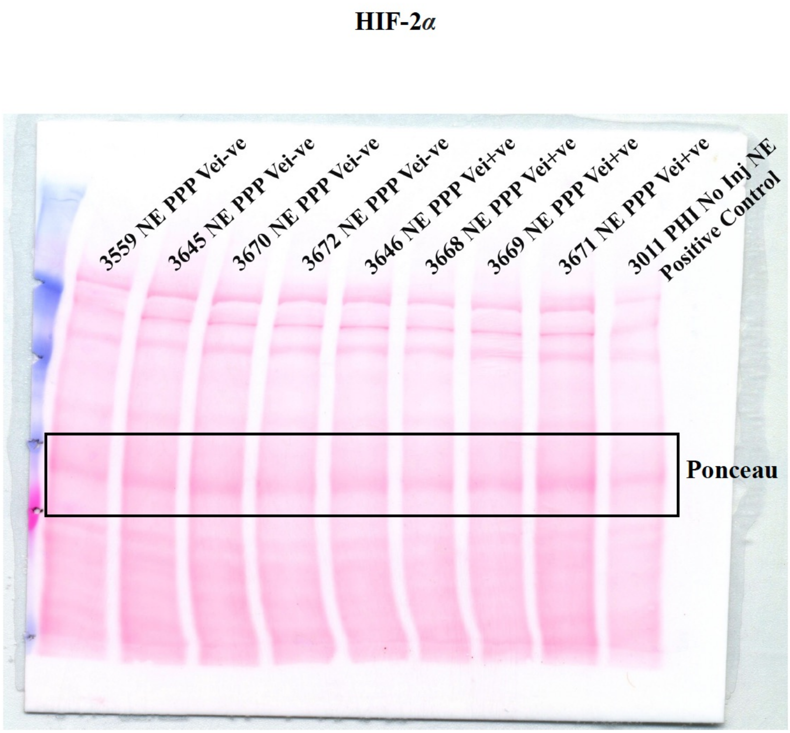


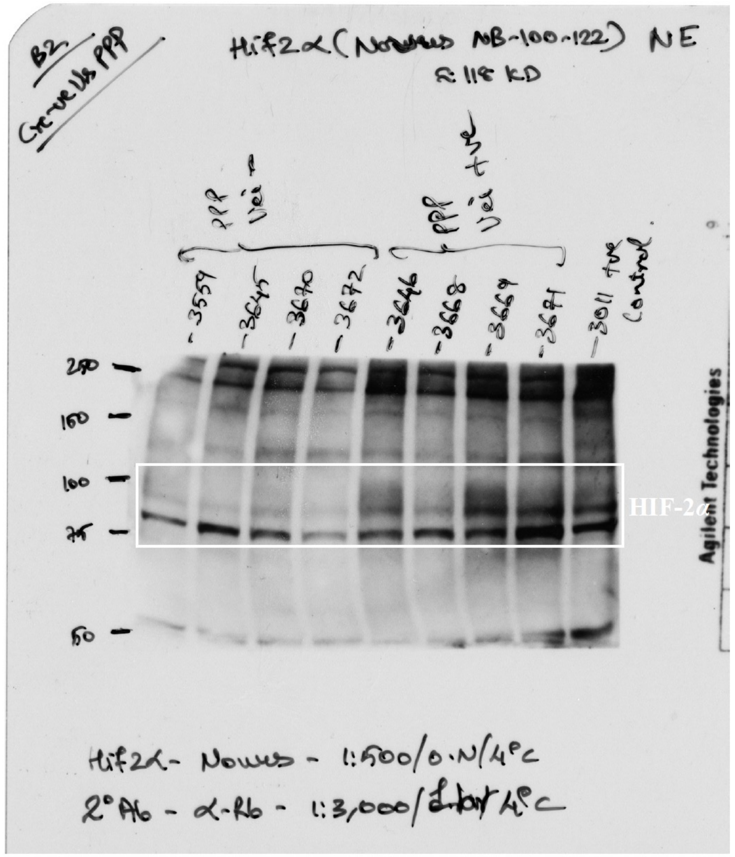

Full unedited gel for Supplemental Figure 14B

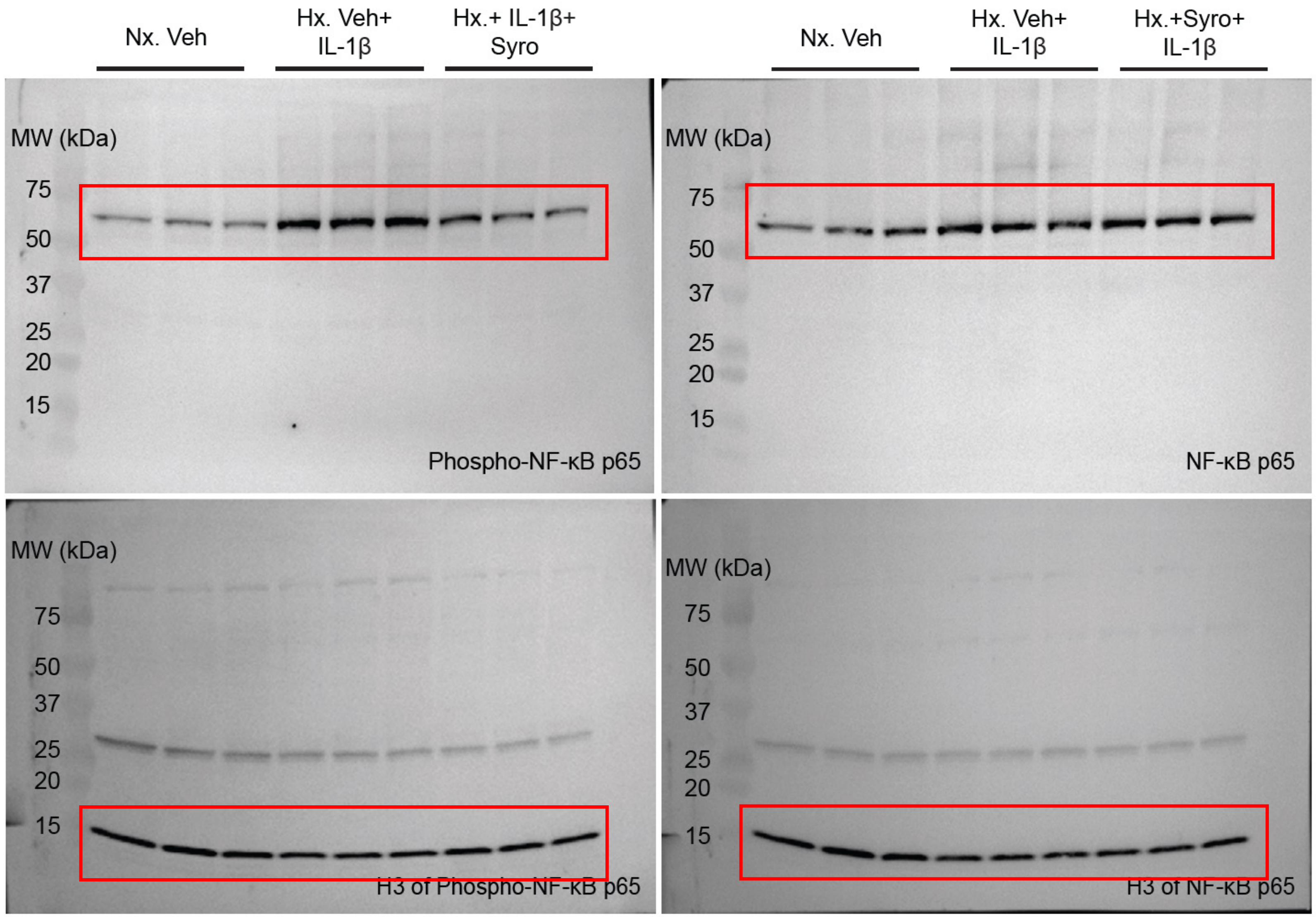


Ladder: Precision plus Protein standards; Cat: 161-0374 (BIO-RAD)
